# Quantitative genome re-sequencing defines multiple mutations conferring chloroquine resistance in rodent malaria

**DOI:** 10.1186/1471-2164-13-106

**Published:** 2012-03-21

**Authors:** Katarzyna Kinga Modrzynska, Alison Creasey, Laurence Loewe, Timothee Cezard, Sofia Trindade Borges, Axel Martinelli, Louise Rodrigues, Pedro Cravo, Mark Blaxter, Richard Carter, Paul Hunt

**Affiliations:** 1 Institute for Immunology and Infection Research, University of Edinburgh, Edinburgh, UK; 2 Laboratory of Genetics and Wisconsin Institute for Discovery, University of Wisconsin-Madison, Madison, USA; 3 The GenePool, University of Edinburgh, Edinburgh, UK; 4 Centro de Malaria e Outras Doenças Tropicais/IHMT/UEI Malaria, Lisbon, Portugal; 5 Centro de Malaria e Outras Doenças Tropicais/IHMT/UEI Biologia Molecular, Lisbon, Portugal; 6 Institute for Evolutionary Biology, University of Edinburgh, Edinburgh, UK; 7 Centre for Immunity, Infection and Evolution, University of Edinburgh, Edinburgh, UK; 8 Current address: The Wellcome Trust Sanger Institute, Hinxton, Cambridge, UK; 9 Current address: Research Unit and Cardiology department, Funchal Hospital Center, Funchal. Madeira, Portugal; 10 Current address: Microbiology, Molecular Genetics and Immunology, Kansas University Medical Center, Kansas City, USA; 11 Current address: IPTSP, Universidade Federal de Goiás, Goiânia, Brasil

## Abstract

**Background:**

Drug resistance in the malaria parasite *Plasmodium falciparum *severely compromises the treatment and control of malaria. A knowledge of the critical mutations conferring resistance to particular drugs is important in understanding modes of drug action and mechanisms of resistances. They are required to design better therapies and limit drug resistance.

A mutation in the gene (*pfcrt*) encoding a membrane transporter has been identified as a principal determinant of chloroquine resistance in *P*. *falciparum*, but we lack a full account of higher level chloroquine resistance. Furthermore, the determinants of resistance in the other major human malaria parasite, *P. vivax*, are not known. To address these questions, we investigated the genetic basis of chloroquine resistance in an isogenic lineage of rodent malaria parasite *P. chabaudi *in which high level resistance to chloroquine has been progressively selected under laboratory conditions.

**Results:**

Loci containing the critical genes were mapped by Linkage Group Selection, using a genetic cross between the high-level chloroquine-resistant mutant and a genetically distinct sensitive strain. A novel high-resolution quantitative whole-genome re-sequencing approach was used to reveal three regions of selection on chr11, chr03 and chr02 that appear progressively at increasing drug doses on three chromosomes. Whole-genome sequencing of the chloroquine-resistant parent identified just four point mutations in different genes on these chromosomes. Three mutations are located at the foci of the selection valleys and are therefore predicted to confer different levels of chloroquine resistance. The critical mutation conferring the first level of chloroquine resistance is found in *aat1*, a putative aminoacid transporter.

**Conclusions:**

Quantitative trait loci conferring selectable phenotypes, such as drug resistance, can be mapped directly using progressive genome-wide linkage group selection. Quantitative genome-wide short-read genome resequencing can be used to reveal these signatures of drug selection at high resolution. The identities of three genes (and mutations within them) conferring different levels of chloroquine resistance generate insights regarding the genetic architecture and mechanisms of resistance to chloroquine and other drugs. Importantly, their orthologues may now be evaluated for critical or accessory roles in chloroquine resistance in human malarias *P. vivax *and *P. falciparum*.

## Background

Despite advances in vector control and attempts to develop effective vaccines, chemotherapy remains a principal mode of malaria control. Unfortunately malaria parasites resistant to drugs such as chloroquine (CQ) have arisen by gene mutation; their prevalence increasing by subsequent selection and transmission. These drug resistant parasites seriously compromise efforts to treat and control malarial disease both in individual cases and in communities. The ability to describe, understand and respond to these evolutionary processes continues to depend upon the identification of the precise genetic mutations which underlie the resistance phenotypes. Also, an identification of the genes involved may lead to insights regarding the mechanisms of drug action and resistance, and the design of improved drugs and treatment strategies.

For chloroquine resistance (CQ-R), genetic linkage studies [1,2], other experimental approaches [3] and phenotype/genotype associations in parasites from natural infections [3-5] have mapped and identified the K76T mutation in the chloroquine resistance transporter, *pfCRT*, as the dominant genetic determinant in the most important human parasite *Plasmodium falciparum*. This protein mediates the export of CQ from the parasite digestive vacuole (DV) [6-8], its presumed site of action [9]. Additionally, specific point mutations in the multidrug resistance gene (*pfmdr1*) encoding an ABC transporter (P-glycoprotein homologue, *Pgh*-1) have also been shown to modulate the level of resistance in CQ-R parasites in transfection experiments [10,11] and in association studies using parasites from natural infections [12,13]. However, these two genes neither account for the full variation of *in vitro *CQ responses, including high-level CQ-R (CQ-hiR) [4,14] nor the appearance of CQ-R in another major human pathogen, *P. vivax *[15].

**Figure 1:**
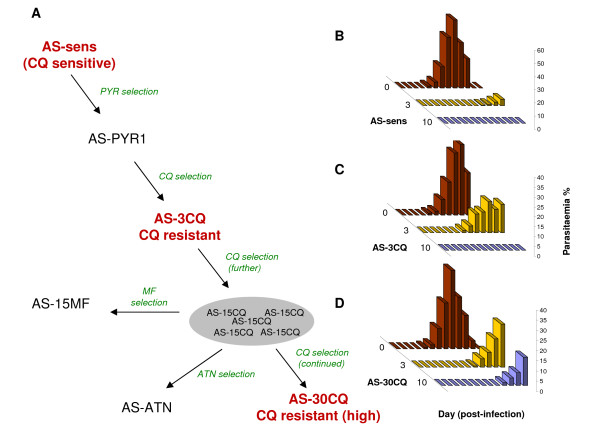
***P. chabaudi *clones in the AS lineage and their chloroquine responses**. **A**. The AS lineage of drug resistant mutants [35,55,56]. Each arrow represents various regimes of selection using drugs (PYR, pyrimethamine; CQ, chloroquine; ATN, artesunate; MF, mefloquine). All strains except AS-15CQ were cloned. **B-D**. The growth of AS-sens (**B**), AS-3CQ (**C**) and AS-30CQ (**D**) at 0 (red), 3 (yellow) or 10 (blue) mg CQ kg^-1 ^day^-1^.

Understanding the genetic basis of CQ-R in the rodent malaria parasite, *P. chabaudi *could illuminate both of these questions; firstly, because, as in *P. vivax*, the orthologues of the *pfcrt *and *pfmdr1 *genes are not involved [16], at least in an existing lineage (Figure 1A) of parasites (strain AS); secondly because this same lineage contains parasites (e.g. AS-sens, AS-3CQ and AS-30CQ) with different levels of CQ-R [17,18]. Previous classical linkage analysis of a genetic cross between the CQ-R mutant AS-3CQ and a genetically distinct sensitive strain, AJ, defined a region of 250 kb on chromosome 11 (chr11) as that containing the mutation conferring the first level of CQ-R [19,20] but the critical gene or mutation was not identified. For *P. chabaudi *CQ-hiR in AS-30CQ, there has been no previous quantitative description of higher level CQ-R or CQ-hiR phenotypes, nor a systematic mapping of the genetic loci containing the critical mutations. Indeed, even the number of genes and mutations involved, and the sizes of their effect have remained undefined [18].

For drug resistance in malaria, classical linkage analysis of genetic crosses has proved effective in mapping single genes conferring clear phenotypes [1-3,21]. Also, Quantitative Trait Loci (QTL) approaches [22,23] or genome wide association studies [24-26] have identified multiple major loci contributing to drug-resistance phenotypes. However, because these methods require the individual characterisation of many parasite lines or clones, they can be time-consuming or may fail to reveal the specific identity of a critical gene. Accordingly, Linkage Group Selection (LGS) [27] or QTL analysis were previously combined with Illumina^® ^whole genome re-sequencing (WGS) to specify the mutations conferring resistance to artemisinin (V2728F *ubp1*) [28,29], sulphadoxine (K392Q *mdr2*) [30], or mefloquine and lumefantrine resistance (*mdr1 *duplication) [31].

Here, we define the basic and high-level CQ-R phenotypes, extend the LGS strategy to map the *multiple *loci contributing to increasing levels of CQ-R, and use *quantitative *WGS of >100,000 single nucleotide polymorphisms (SNPs, differentiating the parental strains of the genetic crosses) to improve their resolution. We propose that, within these loci, mutations occur in the *P. chabaudi *AS-lineage that confer CQ-R and CQ-hiR. We identify these and additional mutations using WGS.

## Results

### The *P. chabaudi *AS lineage contains parasites with increasing levels of CQ-R

In order to quantitate the CQ-R phenotypes in the AS lineage, the clones AS-sens, AS-3CQ and AS-30CQ [17,18] (Figure 1A) were passaged in mice treated with either 0, 3 or 10 mg CQ kg^-1 ^day^-1^. The growth of these parasites (Figure 1B-D) demonstrated that there is an increasing level of resistance to CQ within the lineage. AS-sens parasites grew only in untreated animals. AS-3CQ grew at 0 and 3 mg CQ kg^-1 ^day^-1 ^but not at 10 mg CQ kg^-1 ^day^-1 ^while AS-30CQ was able to survive 10 mg CQ kg^-1 ^day^-1^. We therefore denoted the CQ responses of these clones as CQ sensitive (CQ-S), CQ-R or CQ-hiR, respectively. These data are consistent with a previous proposal that multiple mutations confer CQ-hiR [18] in this lineage, and suggest a suitable range of CQ doses for dissecting the critical genetic loci in LGS experiments, below. For example, we expected that parasites surviving 3 mg CQ kg^-1 ^day^-1 ^would be enriched with parasites having CQ-R (and, possibly, CQ-hiR) phenotypes, while those surviving 10 mg CQ kg^-1 ^day^-1 ^would be preferentially enriched with CQ-hiR parasites only.

### Improved LGS strategies resolve multiple large-effect genes

In the case of drug resistance, LGS uses drug treatment to select the uncloned progeny of a genetic cross (between a drug-resistant clone and a genetically different drug-sensitive parasite) before measuring the proportions of parental alleles in the surviving parasites [32]. It generates a genome-wide scan of selection, revealing 'selection valleys' that are regions of the genome where the proportion of alleles from the drug-sensitive parent is greatly reduced (in drug-treated parasites relative to untreated parasites) and where the genes conferring resistance are located.

In the present study, an uncloned backcross (AS-30CQ × AJ) between the CQ-hiR clone AS-30CQ and the genetically different CQ-S parasite, AJ was generated and treated with different CQ doses (0, 1.5, 3, 10 or 20 CQ kg^-1 ^day^-1^, day 0-2 post-inoculation) to map progressively the signatures of increasing CQ selection. Firstly, the proportions of parental alleles in *all *populations were measured in the surviving parasites, using a library of ~96 pyrosequencing assays [33] (LGS-pyro). Secondly, we developed a novel approach to improve the resolution and confidence of LGS mapping (see Methods, Additional File 1 (section 1)), thus. We defined an expanded set of genome-wide parental AS/AJ SNPs by WGS of the sensitive parent AJ (Additional File 2). 50-base paired-end reads (103-fold mean coverage) were mapped against the 18.8 Mb Wellcome Trust Sanger Institute (Hinxton, Cambridge, UK) AS reference sequence (AS-WTSI). 92% of the reads mapped uniquely. By filtering, we identified 104,667 high stringency SNPs in AJ relative to AS-WTSI at a mean frequency of ~0.0056 substitutions/nucleotide, similar to previous estimates of genetic diversity between the parental strains [34]. At these SNP positions, by counting short sequencing reads containing the AS or AJ base variant in populations of the LGS parasites surviving 0 or 3 mg CQ kg^-1 ^day^-1 ^(88-fold mean coverage for both), we quantitated the proportions of AS and AJ alleles, and investigated (for each SNP) the statistical significance of the difference between the allele proportion after each of the two treatments.

The proportions of alleles (genome-wide) in the LGS populations surviving 3 mg CQ kg^-1 ^day^-1^, revealed by LGS-Illumina and by LGS-pyro were remarkably similar (Additional File 3) suggesting that the experimental errors incurred by either methodology were small.

**Figure 2:**
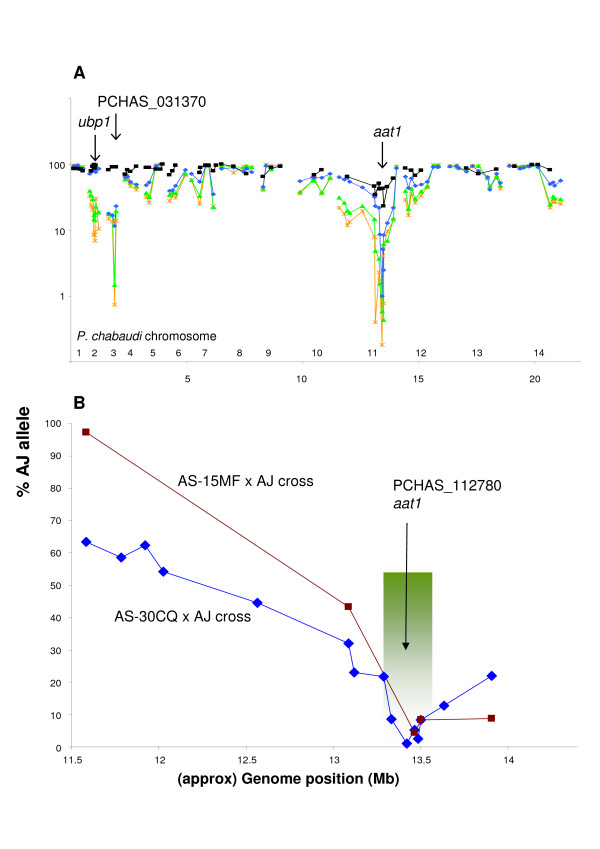
**Scans of chloroquine selection (LGS-pyro)**. Allele proportions (sensitive strain, AJ) in uncloned progeny of genetic crosses using AS/AJ SNPs (pyrosequencing). **A**. Genome-wide - AS-30CQ × AJ parasites surviving 1.5 (black, ■), 3 (blue, ♦), 10 (green, ▲) or 20 (orange, **+**) mg CQ kg^-1 ^day^-1^. The positions of mutations in *aat1*, PCHAS_031370 and *ubp1 *are indicated, and the proportions of the wild-type (AJ) base at these positions (as estimated by proportional sequencing [54]) are included. **B**. Chromosome 11 selection valley - parasites surviving 3 mg CQ kg^-1 ^day^-1^, with position of *aat1 *mutation indicated; AS-30CQ × AJ backcross (blue, ♦), AS-15MF × AJ backcross (red, ■). The region previously defined by classical genetic linkage analysis [20] is shown (gradient shaded green box).

**Figure 3:**
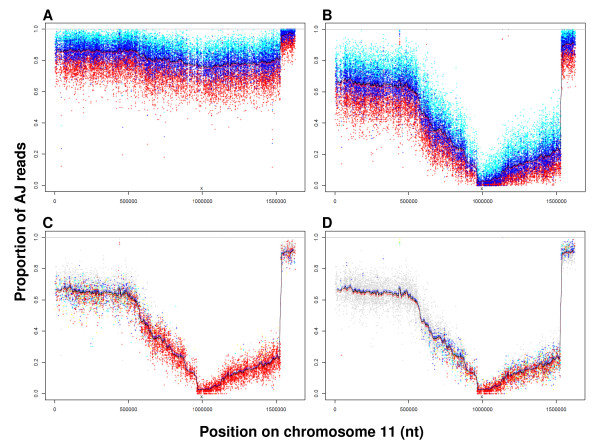
**LGS-Illumina reveals selection by chloroquine on chr11**. Allele proportions (sensitive strain, AJ) in uncloned progeny of genetic crosses using high-stringency quantitative AS/AJ SNPs. The position of *aat1 *point mutation is indicated at bottom of each panel, **x**. **A**. In the absence of CQ, each dark blue cross denotes AJ SNP frequency at each AS/AJ SNP. The upper and lower binomial 95% confidence intervals for this proportion are given in cyan and red, respectively. The black line averages the focal SNP with 50 SNPs on each side (95% confidence interval (CI) = red, blue line). The frequent small changes of mean allele frequencies on a local scale are more likely to reflect stochastic effects rather than 'real' effects of selection. **B**. As "**A**" but after growth in the presence of 3 mg CQ kg^-1 ^day^-1^. **C **As "**B**" without CI of individual SNPs. Colour coding denotes probability that the observed allele frequency in the selected sample is significantly different from that in the unselected sample in "A"; red P < 10^-12^, yellow P < 10^-10^, cyan P < 10^-8^, blue P < 10^-6^, and grey represents other points. **D **As **C**, but with 25% AF-reduction (statistics performed with AJ frequencies (unselected population in "**A**") are reduced by 25%, see Methods). For **A **- **D**, corresponding high-resolution pdf files enable detailed inspection of individual SNPs, sliding-window means and standard errors, available from corresponding author on request.

**Figure 4:**
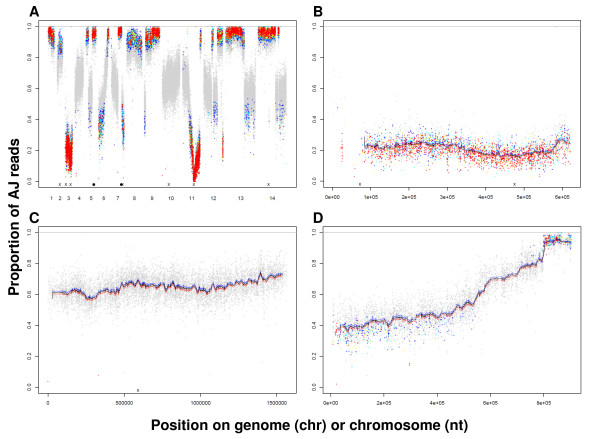
**LGS-Illumina - genome-wide scans of chloroquine selection**. Datapoint colours encoded as in Figure 3D; **A **whole genome, **B **chr03, **C **chr10, **D **chr06. Point mutations and indels are indicated by **x **or filled circles, respectively, at the base of scans, as determined by independent WGS of AS strains AS-sens, AS-30CQ. Corresponding high-resolution pdf files enable detailed inspection of individual SNPs, sliding-window means and standard errors, available from corresponding author on request.

LGS-pyro revealed progressively distinct selection valleys on chr11, chr03 and chr02 as the CQ dose increased (Figure 2A). LGS-Illumina confirmed selection valleys on chr11 (Figure 3A-D, Figure 4A) and chr03 (Figure 4A, B) at 3 mg CQ kg^-1 ^day^-1^. These data suggest that CQ-R phenotypes in the AS lineage are conferred by the action of a major effect gene on chr11, confirming previous linkage analysis [19,20], and for CQ-hiR, major effect genes on chr03, and chr02.

### Mutation in aminoacid transporter (A173E *aat1*) is predicted to confer CQ-R

LGS-pyro identified a partial selection valley on chr11 at 1.5 mg CQ kg^-1 ^day^-1 ^which proved to be dominant at 3 mg CQ kg^-1 ^day^-1 ^(Figure 2A): For example, the proportion of an AJ allele of marker pcpf06-1338 decreased from 69.2% in untreated infections to 2.5% under CQ selection. Importantly, an independent genetic backcross between the mefloquine- and CQ-resistant clone AS-15MF [35] (in the same AS lineage, Figure 1A) and AJ also showed a similar distinct selection valley on chr11 at 3 mg CQ kg^-1 ^day^-1 ^(Figure 2B). Both selection valleys coincided with the 250 kb region previously mapped by classical linkage analysis [20] (Figure 2B).

LGS-Illumina confirmed the selection valley (at 3 mg CQ kg^-1 ^day^-1^) on chr11 at high resolution and statistical significance (Figure 3A-D). Here, a region at nucleotide ~1,000,000 where the proportion of AJ alleles reached a minimum < 3%, was flanked on either side by regions of increasing AJ-allele proportion. The gradual and regular change in AJ allele proportion suggested the presence of many independent recombinant clones in the cross progeny. The differences in the gradients of the two slopes forming the selection valley may reflect different local recombination rates along the chromosome. These data resolved the presence of a mutation that confers CQ-R, close to nucleotide 1,000,000 on chr11. This locus corresponds to the base of the selection valley as defined by LGS-pyro (Figure 2A, B) and to the 250 kb locus previously mapped [20], confirming that the gene bearing the mutation conferring CQ-R lies toward the right-hand end of chr11.

WGS (Methods, Additional File 1 (section 1)) identified a total of 7 point mutations (confirmed by dideoxysequencing) in AS-30CQ relative to AS-sens (Table 1, Table 2, Additional File 4), four of which are shared between AS-30CQ and AS-15MF [29]. Of these four mutations, only one maps to chr11; a non-synonymous mutation (A173E) in a gene (PCHAS_112780) encoding a predicted aminoacid transporter (*aat1*). It is found at base 996,332 (Sanger Sept2009 assembly) coincident with the floor of the chr11 selection valley (Figure 2B, 3B). We concluded that the probability of failing to identify a genuine point mutation (false negative) in this region is very small, for three reasons. Firstly, > ~ 96 - 98% of the AS-WTSI genome was covered by uniquely mapping short-reads (36 - 41 bp) employed here [29] (theoretical maximum ~ 98.5%). Secondly, the read coverage is high: for 200 kb upstream and downstream of *aat1 *on chr11, only 0.61% or 0.73% of bases showed a read coverage of < 5 or < 10, respectively (Additional File 5). Thirdly, we identified a very low overall genome-wide substitution frequency (7 point mutations/genome) in AS-30CQ (Table 1, Table 2) relative to AS-sens.

**Table 1:** Phenotype and Genotype of Selected Clones of the AS *P.chabaudi *Lineage

		gene abbreviation, chromosome, residue										
		***dhfr***^***1***^		*aat1^1^*	**031370**^***2***^		*cir^1^*	*ubp1^1^*	**101550**^***2***^			
		chr07		chr11	chr03		chr03	chr02	chr10	chr14	chr07	chr05
		106		173	102	719	707	2728	162	intergenic	intergenic	
Clone or Strain	PYR response		CQ response									
AJ	Sensitive	S	Sensitive	A	I	T	T	V	Y	wt	wt	wt
AS-sens	Sensitive	S	Sensitive	A	I	T	T	V	Y	wt	wt	wt
AS-PYR	Resistant	N	Sensitive	A	I	T	T	V	Y	mut	34 bp deletion	wt
AS-3CQ	Resistant	N	Resistant	E	I	T	T	V	Y	mut	34 bp deletion	> 1 kbp deletion
AS-15CQ	**this line is uncloned**^***3***^ ^***3***^											
AS30CQ	Resistant	N	Hi-Resistant	E	I	N	N	F	H	mut	34 bp deletion	> 1 kbp deletion
AS-15MF^***4***^	Resistant	N	Resistant	E	del^***4***^	T	T	F	Y	mut	34 bp deletion	> 1 kbp deletion

The pyrimethamine (PYR) and CQ responses of clones of the AS lineage are shown along with all mutations detected in AS-30CQ (and other clones) relative to AS-sens. ^1 ^dhfr, dihydrofolate reductase; aat, aminoacid transporter; cir, chabaudi interspersed repeat; ubp, ubiquitin specific protease (de-ubiquitinating enzyme). ^2 ^Refers to genes PCHAS_031370 or PCHAS_101550. ^3 ^mixed genotype and phenotype, see Additional File 1. ^4 ^Note that AS-15MF carries an alternative allele of PCHAS_031370, namely I102del. Single-letter amino acid code used: A, alanine; E, glutamate; F, phenylalanine; H, histidine; I, isoleucine; N, asparagine; S, serine; T, threonine; V, valine; Y, tyrosine; del, codon deleted.

**Table 2:** Illumina^® ^whole-genome re-sequencing - Confirmed and High-Confidence Mutations in AS-30CQ

Chr		Nucleotide number	WTSI	AS-30CQ	Quality Score	Mutation	Gene (abbrev)	Nearest Gene	*P. falciparum *orthologue
	**POINT MUTATIONS**								
2		216,954	C	A	99	V2728F	PCHAS_020720		PFA0220w
3		70,553	G	T	99	T707N	PCHAS_030200		none
3		474,123	C	A	99	T719	PCHAS_031370		PFB0675w
7		994,546	G	A	99	S106N	PCHAS_072830		PFD0830w
10		634,932	T	C	99	Y162H	PCHAS_101550		PF14_0279
11		996,332	G	T	99	A173E	PCHAS_112780		PFF1430c
14		936,945	T	G	92			PCHAS_142600	PF08_0081
	**DELETIONS**								
	Start (nucleotide number)	Finish (nucleotide number)							
5	683,724	684,989					PCHAS_051920		none
7	876,909	876,914						PCHAS_072420	PF08_0067

Summary of the high confidence mutations proposed in clone AS-30CQ (see Additional File 1). Read depth and quality scores fro mutations were according to SSAHA2. Start and Finish nucleotides for deletions are approximate only.

Dideoxysequencing confirmed that the A173E *aat1 *mutation first appeared in the AS lineage in AS-3CQ, along with the CQ-R phenotype (Table 1).

We therefore propose that *aat1 *A173E is the determinant of CQ-R in this particular *P. chabaudi *lineage.

**Figure 5:**
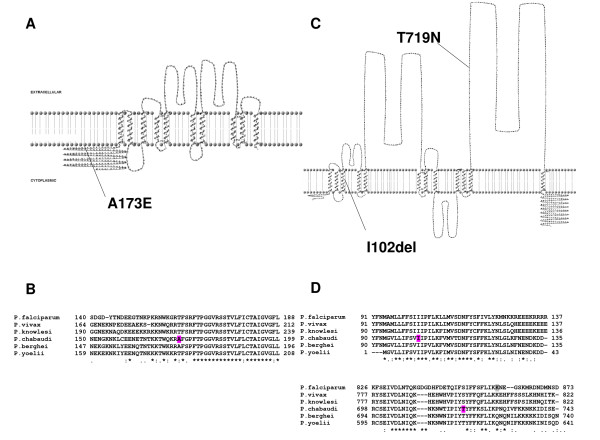
**Structure and sequence conservation of *P. chabaudi AAT1 *and PCHAS_031370 and their orthologues**. Secondary structure predictions of *AAT1 *(**A**) and PCHAS_031370 (**C**) proteins reveal 10 and 12 TM-helix proteins, respectively. The mutations discovered within CQ-R and CQ-hiR *P. chabaudi *parasites (AS-3CQ and AS-30CQ, respectively) are highlighted (magenta). The alignments of *Plasmodium *spp. protein fragments (**B, D**) indicate the positions of mutations in, or close to, conserved regions.

The A173E *aat1 *mutation shares some properties with the determinant (K76T *pfcrt*) of CQ-R in *P. falciparum*. For example, like *pfcrt*, *aat1 *is predicted to encode a 10-transmembrane (TM) helix transporter (Figure 5A) and its *P. falciparum *orthologue (PFF1430c) is targeted to the membrane of the DV (D. Fidock, P. Moura, pers comm.). The wild-type function of *pfcrt *is uncertain but amino acid transport has been suggested [36,37]. Both K76T and A173E mutations result in negative charge shifts. Residue 173 in *aat1 *is at the start of a highly conserved region (Figure 5B) close to the start of the first TM-helix (TM1): in *pfCRT*, residue 76 lies at the start of TM1, predicted to be internal to the DV where CQ is thought to act. These data suggest that *AAT1 *and *CRT *may share some structure/function relationships impacting on their physiological function in the absence and/or presence of CQ.

### Mutation in another transporter (T719N PCHAS_031370) is predicted to confer intermediate CQ-R

LGS-pyro experiments showed that AS markers on chr03 were selected at 3, 10 or 20 but not at 1.5 mg CQ kg^-1 ^day^-1 ^(Figure 2A). On chr03, the proportion of the AJ allele of marker pcpf02-0452 decreased from 79.3% (untreated) to 17.0% at 3 mg CQ kg^-1 ^day^-1^. LGS-Illumina analysis confirmed that AJ alleles are reduced across the whole of chr03; from about 82% in untreated parasites to about 16% in CQ-treated population (Figure 4B). Details of the selection profile on chr03 are consistent with a focus of selection close to base ~480,000. The gradients of this selection valley are not dissimilar to those observed on chr11 when observed on a genome-wide scale (Figure 4A).

WGS of AS-30CQ revealed (Table 2) a non-synonymous mutation (T719N, PCHAS_031370) at base 474,123 on chr03, and dideoxysequencing confirmed that this mutation arose between AS-3CQ and AS-30CQ (Table 1,). However, this mutation does not appear in clones AS-15MF and AS-ATN (Figure 1A, Additional File 6) - these two clones being selected from AS-15CQ (non-clonal) using mefloquine and artesunate, respectively. Instead, AS-15MF and AS-ATN bear a 3 base deletion (I102del) in the same gene (confirmed by dideoxysequencing). We therefore suggest that both the T719N and I102del mutations were partially selected by intermediate levels of CQ in AS-15CQ prior to fixation during treatment with CQ (AS-30CQ), mefloquine (AS-15MF) or artesunate (AS-ATN) and subsequent cloning (Additional File 1, section 3).

PCHAS_031370 is predicted to encode a 12 TM-helix protein (Figure 5C) and its *P. falciparum *orthologue PFB0675w is also predicted (but not yet experimentally confirmed) to target the DV membrane (D. Fidock, P. Moura, pers comm.). The T719N substitution occurs in a large loop between TM11 and TM12, a highly conserved region of the gene (Figure 5D). The I102del mutation is predicted to locate to the centre of TM3 and to change the overall prediction of TM domain structure (data not shown). The general transmembrane domain structure and DV localisation of PCHAS_031370 are therefore similar to other proteins (*pfcrt*, *pfmdr1*, *aat1*) identified as conferring or modulating CQ-R in *P. falciparum *or *P. chabaudi*. These data suggest that the PCHAS_031370 T719N mutation confers an increased CQ-R phenotype.

Interestingly, the *P. yoelii *orthologue (PY05194) of PCHAS_031370 lacks sequence corresponding to TM1 and TM2 that are otherwise present in other *Plasmodium *spp. (Figure 5D). Wild-type *P. yoelii *(17X) had not been exposed to antimalarial drugs but was reported to be highly CQ-R [38]. We suggest the possibility that *P. yoelii *CQ-R resistance might be related to this structural variation.

WGS of AS-30CQ also revealed a non-synonymous mutation T707N in PCHAS_030200 at base 70,553 on chr03 (Table 1, Table 2), predicted to encode a member of the *P. chabaudi*-specific variant gene family (chabaudi interspersed repeat, *cir*) [39]. This mutation maps to the left-hand of chr03. The detailed LGS-Illumina profile of AJ allele proportion (Figure 4B) supports the possibility that this mutation too may contribute to an augmented (intermediate) CQ-R. Dideoxysequencing confirmed that this mutation is specific to AS-30CQ and does not appear in either AS-15MF or AS-ATN.

### Mutation in de-ubiquitinating enzyme (V2728F *Ubp1*) is predicted to confer the highest level CQ-R

LGS-pyro shows that AS markers on chr02 are selected at 10 and 20, but not at 0, 1.5 or 3 mg CQ kg^-1 ^day^-1 ^(Figure 2A): e.g. at 10 mg CQ kg^-1 ^day^-1 ^on chr02, the percentage of AJ alleles of marker pcpf01-0158 decreased from 89.8% (untreated) to 14.0%. Further resolution within the chromosome was not possible because parasites surviving 10 or 20 mg CQ kg^-1 ^day^-1 ^were not analysed by LGS-Illumina. WGS had previously identified a single mutation (V2728F *ubp1*, formerly V770F [28]) on chr02 [29] in both AS-30CQ and AS-15MF. It is the only mutation detected on chr02 and occurred between AS-3CQ and AS-15CQ (during CQ selection, Additional File 1 section 2). We conclude therefore that *ubp1 *V2728F confers CQ-hiR. This mutation was predicted to reduce the activity of a de-ubiquitinating enzyme [28] and also to confer artemisinin resistance in AS-30CQ, without previous exposure to this drug [29]. It is therefore predicted to affect the responses of malaria parasites to multiple drugs with diverse chemical structures and modes of action.

Dideoxysequencing confirms that this mutation appears in AS-30CQ and AS-15MF but not in AS-ATN. Instead an alternative mutation V2697F (formerly, V739F) *ubp1 *appears in AS-ATN [28]. As with the alternative mutations in PCHAS_011370, we suggest that these two alternative *ubp1 *mutations are partially selected (by CQ treatment) in the uncloned parasite AS-15CQ. Their differential selection and fixation in clones AS-30CQ, AS-15MF and AS-ATN derived from AS-15CQ after selection by CQ, mefloquine or artesunate are fully discussed along with a complete resolution of apparent contradictions regarding their linkage (or otherwise) with alternative mutations in PCHAS_031370 (Additional File 1, section 3).

### Other mutations in AS-30CQ

Nine mutations are identified in AS-30CQ relative to AS-sens; seven point mutations and two deletions (Table 1, Table 2).

Four point mutations (on chr11, chr03 (two) and chr02) are associated with signatures of CQ selection and were discussed above. They all first appeared in the *P. chabaudi *AS lineage (Figure 1A) in AS-3CQ or AS-30CQ (i.e. during CQ selection).

A fifth point mutation was identified in AS-30CQ, as predicted, on chr07. This mutation, S106N *dhfr *(encoding dihydrofolate reductase) was confirmed by dideoxysequencing to have first appeared in AS-PYR1. It was previously shown to confer resistance to pyrimethamine [30,32,40].

Four mutations (two point mutations and two deletions), identified in AS-30CQ, were not associated with signatures of drug-selection. Three were confirmed by dideoxysequencing; a non-coding point mutation on chr14, a 34 bp non-coding deletion on chr07 (Additional File 7) (both first appearing in AS-PYR1) and a non-synonymous point mutation on chr10, namely Y162H PCHAS_101550 (orthologue of *P. falciparum *PF14_0279) arising first in AS-30CQ (i.e. during CQ selection). A fourth mutation could not be confirmed by dideoxysequencing: extensive low or zero-coverage and/or a small cluster of poor quality SNP calls in AS-30CQ, (also in AS-15MF [29] but not in AS-50S/P [30] strongly suggested a ~1 kb deletion on chr05 occurring first in AS-3CQ or AS-15CQ (i.e. during CQ selection, Figure 1A). Other studies will be required to evaluate whether these 2 point mutations and 2 deletions are consistently neutral (and consequently randomly fixed during cloning), or whether they play a minor role in drug (pyrimethamine or CQ) resistance. Such roles could include a weak selective advantage in the presence of drugs or compensation for fitness costs incurred by the 'drug resistance' mutations (for example, in the absence of drugs or during transmission of parasites through mosquitoes).

The low probability of failing to identify point mutations (false negatives) on chr11 was discussed in the *AAT1 *section above. Similar arguments and data may be applied equally to the whole genome (Additional File 5) and are addressed more fully here (Additional File 1) and previously [29]. Our conclusion is that the probability of a false negative point mutation in central regions of a chromosome is low (< 0.05). For regions of chromosomes closer to the telomeres where *P. chabaudi*-specific genes are located, we suggest that the probability of a false negative is higher but not easily quantified. However, with the exception of possible selection at the left hand end of chr06, our experiments show no evidence of CQ selection in these regions.

### Genome-wide scan of selection - other observations

Both LGS-pyro and LGS-Illumina data indicated that AJ allele proportions were high (~90%) in the untreated LGS population but were reduced after drug treatment (Figure 2A, 4A) at many loci genome-wide, including chromosomes other than chr02, chr03 or chr11. For example across chr10 (Figure 4C) AJ allele proportions were reduced from ~86% to ~65%, after drug treatment (3 mg CQ kg^-1 ^day^-1^). These data may reflect high AJ proportions in the backcross and the loss of AJ parental parasites (present in a significant proportion) after CQ treatment. Additionally, or alternatively, AJ alleles may have been positively selected during growth without drugs, reflecting the possible action of multiple (small effect) genes that underlie the faster growth of AJ compared to AS parasites, observed routinely in previous experiments [41,42].

The LGS-pyro data showed that the selection valleys on chr11, 03 and 02 were produced progressively at increasing CQ doses (Figure 2A). Thus, low doses resulted in the selection of AS alleles on chr11, and increasing doses resulted in selection of AS alleles on chr03 and then on chr02. We note that the maximum depth of the chr11 selection valley was reached at a lower CQ dose than that required to achieve maximum selection at chr03 (and additionally for chr02). These data may be interpreted by invoking two possible factors. Firstly, we suggest that the mutations conferring CQ-hiR on chr03 and on chr02 may incur 'fitness costs': *i*. *e*. that in the absence of a sufficiently high concentration of CQ, these mutations may *reduce *the growth of parasites. This would mean that, at lower CQ concentrations, parasites with CQ-R (bearing only the 173E *aat1 *allele) would be selected to a greater degree than CQ-hiR parasites bearing multiple mutations. Secondly, the effects of the mutations on chr03 and/or chr02 may be epistatic to the A173E *aat1 *mutation, because mutated AS alleles at these loci (chr03 and chr02) only show signs of selection (at higher doses of CQ) *after *the selection of mutated AS alleles at the *aat1 *locus (chr11) (at lower doses of CQ). According to this interpretation, parasites bearing only the mutations on chr03 or chr02 (or both) are not selectable by lower doses of CQ.

The LGS-Illumina analysis revealed an abrupt discontinuity of AJ proportion at the right hand end of chr11 (Figure 3A-D) and similar changes on chr05, 07, 09, 12, 13 and 14 (Figure 4A, Additional File 8), often in both untreated and drug-treated parasites. These are described and discussed in Additional File 1, section 4. These discontinuities are also observable in the LGS-pyro data. Our conclusion is therefore that they are not artefacts of LGS- Illumina. Also, they did not arise by natural genetic or selection phenomena. They are most likely to arise from differences (in genome assembly) between AS-WTSI (reference strain) and our parental strains AS-sens and/or AJ.

LGS-pyro and LGS-Illumina revealed regions showing possible weak drug selection but where mutations were *not *detected; for example, the left-hand end of chr06 (Figure 4D). Further studies are required to investigate whether these represent reproducible regions of selection or arise from random variation.

## Discussion

We have described *in vivo *CQ-R and CQ-hiR phenotypes in the genetically related *P. chabaudi *AS parasites AS-3CQ and AS-30CQ, mapped the underlying genetic loci on chr11 (CQ-R), chr03 and chr02 (CQ-hiR) and, by WGS, have identified a small number of mutations proposed to confer these phenotypes. A173E *aat1 *(on chr11) is proposed to confer CQ-R. T719N PCHAS_031370 (transporter on chr03) and V2728F *ubp1 *(on chr02) are proposed to confer CQ-hiR. We also suggest that T707N PCHAS_030200 (*cir *gene on chr03) may also contribute to CQ-hiR.

### Mutations conferring CQ-R are identified by integrating genetic and genomic data

The genetic (LGS-pyro and LGS-Illumina) and genomic (WGS) approaches employed showed a remarkable concordance on chr02, chr03 and chr11: *i.e*. these major selection valleys contain 4 of the 6 mutations that arose in the lineage under CQ selection (from AS-PYR1 to AS-30CQ). The genome-wide scans do not reveal selection around the other two mutations arising during CQ selection (Y162H PCHAS_101550, chr10; > 1 kb deletion, chr05) nor around those (three) arising during previous pyrimethamine selection (in AS-PYR1, Figure 1A); *i.e*. 34 bp deletion and S106N *dhfr*) (both chr07), non-coding point mutation (chr14).

The correspondences between the genetic and genomic data and the arguments deployed previously [29] and above (Results - other mutations in AS-30CQ) together suggest that only 3-4 major effect genes conferring CQ-R and CQ-hiR were fixed by strong selection (and cloning) during experimental evolution from AS-sens to AS-30CQ. Furthermore, only a small number of weakly selected or effectively neutral mutations were fixed stochastically during population bottlenecks such as those occurring during transmission of parasite lines, or experimental cloning.

Previous similar investigations in the *P. chabaudi *AS lineage demonstrated that one mutation conferred resistance to each of pyrimethamine, sulphadoxine [30], mefloquine [31] and artemisinin [29] and that ≤ 3 mutations were fixed stochastically each time during the selection and cloning of AS-50S/P, AS-15MF and AS-30CQ respectively. The low rate of nucleotide substitution, even during multiple passages and bottlenecks, makes this system well adapted for investigations of resistance to other drugs.

### The genetic architecture of CQ-R

CQ-hiR was previously generated in AS-30CQ of the *P. chabaudi *AS-lineage by drug selection over about 40 passages [17,18], leading to the suggestion that the CQ-hiR phenotype arose by a series of small steps and was mediated by several mutations at different loci, although the number of mutations was not predicted. Actually, for CQ-R, the present data strongly support the contribution of *aat1 *(chr11) as a single major effect gene, confirming previous classical genetic linkage analysis [19,20]. For CQ-hiR, two (or three) major effect genes (PCHAS_031370 (transporter) and *ubp1*, on chr03 and chr02, respectively) are strongly supported. In this respect, the architecture of CQ-R in *P. chabaudi *may be similar to that of *P. falciparum *where *pfcrt *and *pfmdr1 *alleles appear to generate high levels of CQ resistance epistatically [12,13]. The hypothesis that CQ-hiR in *P. chabaudi *is mediated by a large number of small-effect mutations is rejected by the data presented here.

### The genetic determinants of CQ-R

The concordance between the phenotypic, genetic and genomic data strongly supports the identification of mutations in *AAT1 *as the key determinant of CQ-R. We have also revealed shared properties of *pfcrt *and *aat1 *and their mutations (K76T, A173E, respectively), such as putative function (amino acid transport), subcellular location, TM-helix topology, type and position of mutation. Genome-wide transcription profiling of the CQ-S *P. falciparum *parasite (strain 106/1) and CQ-R transfectants identified the *aat1 *orthologue PFF1430 as one of a small number of differentially expressed genes [37] which also included genes predicted to encode transporters, proteases and components of the trafficking pathways. This suggests future transfection and functional studies to address questions regarding the role of these mutations in amino acid or CQ transport and CQ-R in *P. falciparum *and *P. chabaudi*. The involvement of *aat1*, another (chr03 encoded) DV transporter and *ubp1 *in CQ-hiR also provoke questions regarding the relationship between haemoglobin digestion, protein turnover, amino acid transport/availability and CQ action, export and resistance phenotypes.,

Interestingly, the V2728F *ubp1 *mutation has now been shown to confer resistance to both artemisinin [29] and chloroquine (this study). This resolves a previously unexplained result - that artemisinin resistance mediated by mutations in *ubp1 *appeared before parasites were exposed to artemisinin. This data also suggests that there must be some commonality in the molecular mechanisms of resistance to the two drugs, at least in the *P. chabaudi *AS lineage.

### The rodent model and public health

*P. chabaudi *and *P. falciparum *have similar or identical genetic bases for resistance to pyrimethamine (point mutations in *dhfr*, [30]) and to mefloquine (*mdr1 *amplification) [31]. For CQ-R, both parasite species share key features (*e.g*. reduced CQ accumulation in DV [43], changes in DV morphology [44] and verapamil reversibility [45]). Now, notwithstanding the evidence supporting the proposal that different genes confer CQ-R in the two species, the data presented here suggest that some features of the molecular mechanism of resistance acquisition may be similar. This supports the use of the rodent model to identify candidate genetic markers of resistance to future antimalarial drugs. Furthermore, the orthologues of *aat1 *(PVX_114575, PFF1430c) and PCHAS_031370 (PVX_002795, PFB0675w) should now be evaluated as candidate molecular markers of CQ-R in *P. vivax *and modulators of CQ responses in *P. falciparum*.

The experiments reported here share some features previously exploited by genome-wide selection scans in yeast [46], genome-wide association [26] and high-resolution identification of mutated genes [47,48] in *P. falciparum*. The present study demonstrates how similar approaches can be used to identify genes conferring complex selectable phenotypes such as drug-resistance in experimental systems.

## Conclusions

Previously, increasing chloroquine resistance phenotypes were experimentally selected in a lineage of the rodent malaria, *Plasmodium chabaudi*. Here, these phenotypes were measured, the underlying genetic loci mapped and mutations specified using a novel quantitative genetics and genomics approach.

This approach analysed genetic crosses by selecting progeny *en masse *at different drug concentrations. The frequencies of parental alleles in the surviving parasites were measured using ~ 100 pyrosequencing single nucleotide polymorphism (SNP) assays, and for ~100,000 single nucleotide polymorphisms, by Illumina short-read sequencing. This defined 'selection valleys' on chromosomes 11, 3 and 2, where genes conferring resistance were expected to be located. Whole genome re-sequencing of the chloroquine resistant mutant parasite and the sensitive progenitor wild-type parasite showed that only 7 point mutations in the whole genome had arisen in the lineage. The specific isolated mutations within the selection valleys were identified. A mutation in a putative aminoacid transporter (*aat1*) encoded on chr11 confers chloroquine resistance. Mutations in another transporter (PCHAS_031370, chr03) and a deubiquitinating enzyme (*ubp1*, chr02) confer higher level chloroquine resistance. Orthologues of these genes in *P. falciparum *and *P. vivax *can now be studied for their contribution to chloroquine resistance in human infection. These data will generate insights of the mechanism of chloroquine resistance in human and rodent malaria parasites.

## Methods

### Parasite and mouse strains, routine passage

AJ and AS-sens are chloroquine (CQ) sensitive clones of the rodent malaria *Plasmodium chabaudi chabaudi*, isolated from wild-caught *Thamnomys rutilans *thicket rats from the Central African Republic [49]. AS-PYR, AS-3CQ and AS-30CQ were selected (and cloned) sequentially from AS-sens by pyrimethamine, CQ and high CQ concentrations progressively and respectively [18,50]. All resistant phenotypes were heritable and stable after cloning, freeze/thaw cycles, passage of parasite without drug, and after transmission through the mosquito host. Six- to eight-week old laboratory CBA female mice were used for all the experiments with the exception of mosquito transmission when C57/BL6 mice were used. All animals were housed and maintained according to the standard animal husbandry conditions, with free access to food (RM3 diet) and PABA supplemented water. All experiments were conducted in compliance with the United Kingdom Animals (Scientific Procedures) Act 1986.

### Drug phenotyping

Mouse infections were initiated with an intraperitoneal inoculum of 10^6 ^parasites and treated with CQ sulphate (Beacon Pharmaceuticals) diluted to appropriate concentration with water and administered to a 20 g mouse by gavage in 100 μl. The treatment was repeated for the first 3 days of infections (d0-2 post-infection (p.i.)). The parasitaemia of all infections was monitored by daily thin blood smears as described previously [29]. Each treatment group consisted of three animals.

### AS-30CQ × AJ backcross and LGS

The AS-30CQ × AJ cross was performed by allowing *Anopheles stephensi *mosquitoes to feed upon anaesthetised C57/BL6 mice infected with mixture of both strains, according to the protocol established previously [29]. After 14-15 days, salivary glands were dissected and sporozoites injected into donor mice. The recovered cross-progeny asexual forms were further passaged, treated with 0, 1.5 or 10 mg CQ kg^-1 ^day^-1 ^for three days, and pooled in order to increase the numbers of resistant recombinants in the mixture. The resulting parasites were backcrossed with the sensitive parent (AJ) using the same procedure as above. The backcross recombinant progeny were selected *en masse *with 0, 1.5, 3, 10 and 20 mg CQ kg^-1 ^day^-1 ^(d0-2 p.i.) in groups of 5 mice each. When parasitaemias within a group reached > 10%, blood was harvested, pooled and DNA isolated using the protocol previously described [34].

### Pyrosequencing

The PSQ™ HS-96A pyrosequencing system was used to measure the proportion of AJ alleles in all selected backcross populations. A set of ~96 uniformly spaced, quantitative pyrosequencing assays measuring the proportion of the SNPs between the AS and AJ were designed as previously described [33]. The pyrosequencing assays were prepared and performed according to the manufacturer's instructions, each assay being performed in triplicate, on three different template samples.

### Genome re-sequencing and mutation detection in AS-30CQ

The chloroquine resistant AS-30CQ genome and that of its sensitive progenitor AS-sens were sequenced with 50 and 36 base single reads at approx 80- and 40-fold coverage, respectively by the GenePool Genomics Facility http://genepool.bio.ed.ac.uk/. The single end reads obtained for AS-30CQ were aligned against the isogenic *P. chabaudi *AS strain reference genome ftp://ftp.sanger.ac.uk/pub/pathogens/P_chabaudi/Archive/September_2009_assembly/ using the MAQ [51]http://maq.sourceforge.net/maq-manpage.shtml and SSAHA2 [52]ftp://ftp.sanger.ac.uk/pub/zn1/ssaha_pileup/ssaha_pileup-readme software suites. The SNPs and indels between the two strains were identified and analysed (Additional File 1, section 1) as previously described [29].

### Quantitative sequencing (LGS-Illumina)

AJ and the AS-30CQ × AJ backcross (surviving 0 or 3 mg CQ kg^-1 ^day^-1^) were each sequenced using ~2 μg of DNA on single lanes, using 50 base paired end reads that were mapped against the AS-WTSI reference sequence (PlasmoDB 6.3 version, 17 Feb 2010) using BWA (v0.5.8) software [53]. For AJ/AS SNP detection, unique reads with mapping quality > 30 and bases with base quality (Phred-like code) > 20 were used. SNPs were called in positions covered by at least 10 reads if at least 30% of the bases are different from the reference.

### SNP selection

113,746 candidate AS/AJ SNPs were determined by Illumina^® ^whole-genome re-sequencing (WGS) of CQ-sensitive parental strain AJ (relative to reference strain sequence AS-WTSI). Of these 838 (0.74%) are in contigs that are currently not assembled in the 14 chromosomes and were ignored in the analysis presented here. For inclusion in the plots presented in this work, the remaining 112,908 SNPs were further filtered using the following criteria: (i) ≥ 20 reads in all samples (i.e. the AJ sequence sample, the untreated cross-progeny sequence sample and the CQ-treated cross-progeny sequence sample, (ii) > 90% frequency of the AJ base-call in the AJ sequence sample (usually this is 100%). SNPs failing these criteria (7,079) were excluded leaving 104,667 SNPs for the genome wide scan of selection. The numbers of AS and AJ nucleotide calls at these SNPs were determined from the sequencing data of AJ and AS-30CQ × AJ (untreated and treated at 3 mg CQ kg^-1 ^day^-1^) samples using custom scripts.

### Statistics - Binomial test

The quantitative LGS-Illumina analysis samples reads from a population of individual clones that are the result of the recombination and selection processes described above. For each SNP the finite sampling size results in random deviations from the expected value for the true allele proportion. These random deviations can be described by the binomial distribution, which is the basis of the tests for statistical significance we employ. As expectation *p *we use the point estimate obtained from our data, where the 'number of successes' *x *are given by the number of AJ alleles observed and the total number of attempts *n *is given by the sum of AJ + AS alleles sequenced (usually equal to the number of reads containing the SNP). We use a two-sided binomial test with a confidence limit of 95% throughout the paper. This means that strong AF-reductions (see below) can result in "significant" points at high AJ allele frequencies, indicating possible selection for AJ alleles.

### Statistics - sliding window analysis

Because the frequencies of individual SNPs come with binomial variability, we sought to estimate the local AJ proportions with reduced sampling error by combining neighbouring SNPs in a sliding window analysis (on the assumption that the real AJ allele frequency was constant over this scale). We summed *x *and *n *for 101 neighbouring SNPs (50 on each side of a focal site) and computed upper and lower limits (using binomial test described above), shown by blue and red lines in Figure 3A-D and Figure 4A-D.

### Statistics - AF-reduction

As described in 'Results' and elsewhere [29], there are factors that may reduce AJ allele proportions at loci not linked to the drug resistant phenotype in drug-treated samples relative to untreated samples. The effects of these factors are very difficult to estimate precisely. We therefore increased the stringency of our statistical analysis by applying a correction parameter, termed "Allele Frequency Reduction" (AF-reduction). This parameter reduces the observed % of AJ alleles in the untreated population (in order to correct for additional and confounding factors, such as the removal of AJ parental parasites). To test the robustness of our conclusions we tested the significance of differences between the drug-treated and the untreated samples with AF-reduction = 0% and 25%. We then computed the probability that our drug-treated samples could have been produced by allele frequencies as observed in the point estimates of our untreated samples. These probabilities are indicated by the colour of each SNP shown (Figure 3C, D and Figure 4A-D). In these plots, we highlight probability P < 10^-6 ^as an important threshold by coloured data points. The choice of this threshold can be justified by considering the large number of SNPs used in this test (approximate Bonferroni correction). At AF-reduction = 25% we found 7342 SNPs that deviated from their expected values with P < 10^-12 ^(red in Figure 3C, D, 4A-D), 3588 with 10^-12 ^< P < 10^-10 ^(yellow), 5945 with 10^-10 ^< P < 10^-8 ^(cyan) and 9272 with 10^-8 ^< P 10^-6 ^(blue). In these SNPs, the AJ-allele proportion was either below or above the expected values with the specified probability. Lower SNP frequencies indicate the valleys of selection caused by resistance mutations. Higher SNP frequencies could indicate the presence of AJ alleles benefiting the growth of parasites in the presence of drugs and other more complex growth, virulence and immunity traits. Alternatively, many significant higher SNP proportions might indicate that the AF-reduction operation was too large.

### The confirmation of predicted mutations

The presence of all mutations predicted between the AS-sens and AS-30CQ clones was confirmed using standard PCR and dideoxysequencing using primers presented in Additional File 9. Additional clones from the AS lineage (AS-PYR, AS-3CQ, AS-15MF, AS-ART and AS-ATN) were also tested, when required, to determine and confirm when mutations arose in the lineage.

### Proportional sequencing

The proportions of mutant alleles at *aat1*, PCHAS_031370 and *ubp1 *genes were measured in untreated and treated populations of parasites using proportional sequencing as described previously [54]. The fragments containing the pre-defined mutations were amplified and sequenced using specific primers (Additional File 9). The resulting electropherograms were analyzed using Chromas 2.33 software (Technelysium Pty Ltd) and the heights of peaks corresponding to the wild-type and mutated nucleotides measured. These were used to calculate an index of the respective proportion of the wild-type allele (AJ) in the population.

### Homology studies and protein structure predictions

The orthologues of mutated genes were identified in other *Plasmodium *spp. using PlasmoDB database and the alignments produced using ClustalW2 software http://www.ebi.ac.uk/clustalw/. The positions of TM-helices in both proteins was predicted using TMpred software http://bioinformatics.biol.uoa.gr/TMRPres2D/ and visualised using TMRPres2D java applet http://liao.cis.udel.edu/website/servers/TMMOD/.

## Abbreviations

AAT1: amino acid transporter 1; ABC: ATP-binding cassette; CQ: chloroquine; CQ-R: chloroquine resistant: chloroquine resistance; CQ-hiR: high level chloroquine resistance; CRT: chloroquine resistance transporter; DV: digestive vacuole; LGS: linkage group selection; LGS-pyro: LGS analysed by pyrosequencing; LGS-Illumina: LGS analysed by quantitative Illumina whole-genome sequencing; Pgh-1: P-glycoprotein homologue-1; QTL: quantitative trait loci; SNP: single nucleotide polymorphism; TM: transmembrane; WGS: whole genome (re-)sequencing; WTSI: Wellcome Trust Sanger Institute.

## Authors' contributions

KM, AC, SB and LR performed the biological and genetic experiments and confirmed mutations. LL, TC analysed and interpreted WGS SNP data in strain AJ, and quantitative LGS-Illumina data. AM analysed WGS for AS mutation detection. PC, MB, RC interpreted and supported research. PH conceived the study and managed the project. KM and PH wrote the paper with assistance from LL, TC and AM. All authors have read and approved the final manuscript.

## Authors information

The authors declare that they have no competing interests.

## Supplementary Material

Additional file 1 **Additional Text**. Section 1, Solexa genome re-sequencing; Section 2, Other mutations in AS-30CQ; Section 3, AS-15CQ and the origins of different haplotypes in subsequent clones; Section 4, Discontinuities in AJ allele frequency. Click here for file

Additional file 2 **(Table) Solexa whole-genome re-sequencing metrics**. Click here for file

Additional file 3 **(Figure) LGS-pyro v LGS-Illumina**. Comparison of genome scans (LGS-pyro (top), LGS-Illumina (bottom)) show near perfect correspondence between the two methodologies. Vertical axis (linear) indicates proportion of AJ alleles in parasites surviving 3 mg CQ kg^-1 ^day^-1^. Horizontal axis indicates chromosome number, top and bottom or genome co-ordinate (Kbase), top only. Position of mutations (AS-30CQ relative to AS-sens) are indicated at bottom of bottom panel (7 SNPs **x**, 2 deletions •). Click here for file

Additional file 4 **(Table) AS-30CQ Genome re-sequencing**. Summary of all the mutations proposed in clone AS-30CQ (Additional File 1). Highlighted are mutations confirmed by di-deoxy sequencing (green), rejected mutations (red), a high confidence deletion (yellow) and low confidence mutations (orange). Read depth according to SSAHA2 is provided for SNPs. All quality scores for SNPs were according to SSAHA2. Small indel quality scores indicate the number of reads calling an indel divided by the total number of reads covering the indel. For large indels and CNVs, a comparative coverage was calculated as described (Methods section and Additional File 1). Click here for file

Additional file 5 **(Table) Genome-wide analysis of % of bases with read-coverage ≥ 10**. Click here for file

Additional file 6 **(Figure) The appearance of mutations in the AS lineage**. Mutations are described by chromosomal location, gene ID, specific amino acid change etc. Some were previously described [29,30] (blue). Novel mutations are identified here (red). For both PCHAS_030137 and *ubp1*, alternative mutations arising between AS-3CQ and AS-15CQ (and individually selected in AS-15MF [31,35] and AS-ATN [55] during mefloquine and artesunate selection, respectively) are defined (*a-d*). Refer to Additional File 1(section 3) for further details. Click here for file

Additional file 7 **(Figure) Chromosome 7 - 34 bp deletion**. Close to the 3' end of gene PCHAS_072420, an alignment of nucleotide sequences for reference genome sequence (AS-WTSI), the sensitive AS lineage progenitor (AS-sens) and the drug resistant mutants AS-PYR, AS-3CQ, AS-30CQ and AS-ART is shown. Symbols: -,34 bp deletion in AS-PYR and subsequent clones (wrt nt 197 - 230 (AS-WTSI arbitrary numbering) inclusive), 15 bp deletion in AS strains (wrt nt 306 - 320 (AJ arbitrary numbering) inclusive); *, nucleotides identical in all clones and strains investigated here (note high frequency of AS/AJ SNPs). 3' end of coding sequence of gene PCHAS_072420 is indicated (upper case, green highlighting); intron (lower case, grey highlighting), 3'-UT and intergenic region indicated (lower case); termination codon (red). Repetitive sequences which may mediate the 34 bp and 15 bp deletions are indicated (yellow) in individual representative clones. Click here for file

Additional file 8 **(Table) Discontinuity co-ordinates**. Co-ordinates represent nucleotide position on chromosome relative to the AS-WTXI sequence assembly (Sanger Sept 2009); nd, cannot be determined. Click here for file

Additional file 9 **(Table) Primers used**. These oligonucleotide primers were used to confirm the predicted mutations in AS lineage. Pairs of primers marked with * were also used for proportional sequencing. Click here for file
