# Supplementary material for: Quantitative genome re-sequencing defines multiple mutations conferring chloroquine resistance in rodent malaria
Source: BMC Genomics. 2012 Mar 21;13:106. doi: 10.1186/1471-2164-13-106 (PMC3362770; doi:10.1186/1471-2164-13-106)
Supplement: Additional file 1 — Additional Text. Section 1, Solexa genome re-sequencing; Section 2, Other mutations in AS-30CQ; Section 3, AS-15CQ and the origins of different haplotypes in subsequent clones; Section 4, Discontinuities in AJ allele frequency. [file 1471-2164-13-106-S1.PDF]

Additional File 1

There are 4 sections

Section 1	Solexa genome re-sequencing
Section 2	Other mutations in AS-30CQ
Section 3	AS-15CQ and the origins of different haplotypes in subsequent clones.
Section 4	Discontinuities in AJ allele frequency in quantitative Solexa and pyrosequencing whole-genome scans of selection

Section 1

Solexa genome re-sequencing

In total, a provisional list of 53 potential mutations was identified: 12 point mutations, 1 CNV, 30 small indels and 10 large indels (Additional File 4).

Eight potential point mutations were investigated by di-deoxy sequencing. Seven (code green) were confirmed, while one was rejected as a false positive call (code red). The confirmed point mutations displayed medium or high coverage (>20 reads) and high SSAHA2 quality scores (lowest SSAHA2 quality score: 92, max = 99). Conversely, the rejected mutation and the four unverified point mutations displayed low SSAHA2 quality scores (highest score 16) and, with one exception, poor coverage (<10 reads). Furthermore, unlike the seven confirmed point mutations, the unverified and rejected mutations were called by SSAHA2 but not by MAQ. For these reasons, these unverified mutations were classified as low confidence mutations (code orange). Three of them were located in unassigned contigs (referred to as "bin" in Additional File 4). These contigs are likely to be less mappable regions and to consist mostly of subtelomeric and telomeric regions of various chromosomes. Due to the likely repetitive nature of these regions, it also proved impossible to design reliable primers for dideoxysequencing sequencing. The remaining unverified point mutation was on a gene on chromosome 5 in a region thought to bear a large deletion (see below). We have observed this phenomenon widely (data not shown) and refer to such SNP calls as 'likely proxies for indels'. They may represent mismapping of reads due to the presence of the deletion. The nucleotide here is also

characterised by very low coverage in AS-sens (data not shown) which may indicate either problems with the generation of reads due to the nature of the sequences in the region or possible assembly errors, emphasising the unreliability of potential calls made in this region.

For small indels, we evaluated a number of those predicted for AS-30CQ and other clones. For instance, here, we investigated two of the higher quality 1 bp deletion calls. Both were rejected (code red) (Additional File 4). We therefore expected that most, if not all, of the other small indel calls to be false positives. However, the verification of a potential 3 bp deletion of intermediate coverage on gene PCHAS_031370 in a different clone within the lineage (AS-15MF, data not shown) by di-deoxy sequencing , raises the possibility that a limited number of other small indels may be identified by future analyses.

For both large indels and CNVs, the following factors were considered; a) whether both MAQ and SSAHA2 identified the mutations, b) visual confirmation of differences in read coverage between AS-sens and AS-30CQ and c) their presence in other sequenced clones of the AS lineage including AS-ART (derived from AS-30CQ, unpublished data). Only a single large indel (a 34bp deletion on chromosome 7) was verified and confirmed by di-deoxy sequencing. One large putative deletion on chromosome 5 was identified by both MAQ and SSAHA2 (code yellow). The remaining large indels were classified as low confidence mutations (code orange). A single potential CNV was predicted on chromosome 14 by SSAHA2 in AS-30CQ only and was therefore classified as a low confidence mutation. These deletions and CNVs could not contribute to CQ-R because these chromosomes were not indicated by the genetic evidence.

Section 2

Other mutations in AS-30CQ

We identified seven point mutations in AS-30CQ (Table 1, Table 2, Additional File 4), proposed by Solexa whole genome resequencing and confirmed by dideoxysequencing. In addition to four specified in this present paper (chr02, chr03 (two) and chr11) associated with CQ selection valleys, we note that S106N in dihydrofolate reductase (*DHFR*, PCHAS_072830) (chr07) confers resistance to pyrimethamine. We also confirmed a non-synonymous mutation on chr10 (Y162H PCHAS_101550, orthologue of *P. falciparum* PF14_0279), showing some sequence similarity to a voltage-gated potassium channel) that arises between AS-3CQ and AS-30CQ, and, on chr14, an intergenic point mutation lying between genes PCHAS_142590 and PCHAS_142600 which arises between AS-sens and AS-PYR.

We also analysed potential Copy Number Variations (CNVs) and insertion/deletion mutations (indels) (Additional File 4). Read coverage analysis detected a 34bp deletion in an intragenic region of chr07 close to the 3' end of PCHAS_072420, appearing first in AS-PYR [22] (Additional File 7). A larger deletion (>1 kb) on chr05 within a *P. chabaudi*-specific gene, PCHAS_051910-20 appeared first in AS-3CQ (n.b. the two IDs are refer to one gene (an annotation artefact) These two point mutations (chr10, chr14) and two deletions (chr05 and chr07) were not associated with drug selection valleys (Figure 4A). Further investigations are required to determine if these mutations are neutral and randomly fixed during cloning or whether they play some role (e.g compensatory, or preparatory) in drug resistance or other growth phenotype.

Section 3

AS-15CQ and the origins of different haplotypes in subsequent clones.

Background

In a previous study, we identified two alternative non-synonymous point mutations (V2697F and V2728F) in *ubp1* (PCHAS_020720) in the AS *P. chabaudi* lineage [24]. These mutations appeared in AS-ATN and AS-30CQ/AS-15MF respectively. These 3 clones all originate from AS-15CQ. These data were interpreted [21] as follows. We noted that AS-15CQ was not cloned [17]. We concluded that both *ubp1* mutations arose independently and were both selected in AS-15CQ by chloroquine treatment.

Interpretation of present data

In this paper, we have identified a similar case where two alternative independent non-synonymous mutations in another gene, PCHAS_031370, occur in AS-30CQ, AS-15MF and AS-ATN. These mutations are T719N in AS-30CQ and I102del in AS-15MF and AS-ATN (main text, Additional File 6). We interpret these data in a similar manner to the conclusions regarding *ubp1* mutations. We conclude that both PCHAS_031370 mutations independently arose and were selected in AS-15CQ by chloroquine. The genetic evidence presented here suggests that these latter mutations (PCHAS_031370) may have arisen before the *ubp1* mutations: *i.e.* the *ubp1* mutations occurred on a mutant PCHAS_031370 background.

We therefore suggest that the V2728F *ubp1* mutation occurred in a 102I, 719N PCHAS_031370 background. This was selected in AS-30CQ by further chloroquine selection and cloning [17]. Similarly, we suggest that the V2697F *ubp1* mutation occurred in a 102del, 719T PCHAS_031370 background. This was selected in AS-ATN by artesunate selection and cloning [47]..

However, a problem arises with the interpretation of the AS-15MF genotype (2728F *ubp1* and 102del, 719T PCHAS_031370 haplotype). This haplotype could arise in two alternative ways.

Firstly, a particular mutation could occur twice in two different genetic backgrounds. For the example discussed above, the 2728F *ubp1* mutation could have occurred in both PCHAS_031370 mutant backgrounds. One parasite would give rise to AS-30CQ and the other to the AS-15MF haplotype.

Secondly, recombination of markers on chr02 and chr03 could have occurred leading to the association of the 2728F *ubp1* allele with the 102del PCHAS_031370 allele in AS-15MF. Indeed, we note that parasites obtained from AS-3CQ (AS-15CQ and beyond) by chloroquine selection were passaged through mosquitoes on more than one occasion [17]. This could lead to re-segregation of unlinked alleles of genes on chr02 and chr03, for example.

This second hypothesis explains all the observed results and is most parsimonious. We note that we have presumed that the PCHAS_031370 mutations occurred (and were partially selected) first and that the *ubp1* mutations occurred on these backgrounds. Furthermore, we have for simplicity discussed the case where 2728F *ubp1* occurs on the 719N PCHAS_031370 background (in AS-30CQ for instance) and 2697F *ubp1* on the 102del PCHAS_031370 background (in AS-15ATN). Other models are possible where the same logical structure is conserved. The example given uses the smallest number of individual mutations and recombination events in the minimum number of clones, and is consistent with the genetic selection data which suggests the PCHAS_031370 (chr03) mutations confer intermediate resistance to chloroquine while *ubp1* mutations (chr02) confer high level resistance.

Section 4

Discontinuities in AJ allele frequency in quantitative Solexa and pyrosequencing whole-genome scans of selection

Inspection of the quantitative Solexa genome-wide scans showed abrupt 'discontinuities' in AJ allele proportion (especially after CQ selection). In these regions, the proportion of AJ alleles rose or dropped suddenly between two adjacent SNPs.

Interestingly, the most notable was that toward the right-hand end of chr11, where AJ allele proportion changed from ~ 0.23 to ~ 0.9. In most cases, discontinuities (at the same locations) were observed in both untreated and drug-treated parasite populations.

Discontinuities are not artefacts of Solexa sequencing

The pattern of discontinuities was compared in the Solexa and pyrosequencing read-outs of LGS (Additional File 3).

The discontinuities on chr05, 07, 09, 11, 12, 13 and 14 in the Solexa data correspond to rather similar abrupt changes in the pyrosequencing data. This remarkable agreement between the datasets confirm the validity of both methods and suggests that the discontinuities reflect real allele proportions in parasites surviving chloroquine treatment, rather than representing artefacts of Solexa sequencing or pyrosequencing.

Next, we discuss further analyses and possible interpretations of these data. A number of interpretations for the discontinuities were considered.

Small number of recombination events.

Do the discontinuities reflect a small number of recombinant parasites in the population? The pre-supposition here is that the discontinuities reflect cross-over events in a limited number of recombinant clones. A discontinuity would signify where a dominant clone (in the population) contains a cross-over. We believe that this is unlikely. One reason is that there are regions of the genome (for example, on chr11 and chr06) where the allele proportions change gradually over a long distance. This implies that there are many recombinant progeny which are well distributed as regards their proportion in the population.

Many recombination events at a small number of hotspots

If there are limited numbers of genome loci with extremely high recombination rates ("hotspots"), and if selection favours different allele frequencies on both sides of such a hot spot, then this could generate extreme discontinuities. However, this explanation would require a combination of highly localised hot-spots and (large-effect) selectable loci causing the discontinuity, while *simultaneously* postulating the absence of such hotspots elsewhere in the genome. Such combinations would have to occur at a small number of different loci where the discontinuities are abrupt, and be absent in those regions of the genome that show smooth selection gradients (e.g. chr11). Furthermore, the recombination rates at these hotspots would need to be extraordinarily large. Although it is beyond the scope of this study to estimate the probability that such a very peculiar distribution of recombination rates and selection coefficients pertains, we believe that this explanation is highly unlikely.

Do the discontinuities reflect differences in genome assembly, synteny or karyotype between parasite clones and strains?

According to one variant of this explanation, discontinuities may reflect a difference between the parental AS and AJ genomes. Discontinuities would represent 'intraspecies (i.e. inter-strain) synteny breakpoints (SBPs)'. Their behaviour during genetic recombination may be difficult to predict but preliminary analysis suggests that gradients of allele proportion would appear on one or other side of the discontinuities (in the presence of selection). As this appears not to be the case for most of the examples, we believe that this interpretation cannot explain the instances where abrupt discontinuities are flanked on both sides by regions of constant AJ proportion.

Alternatively, discontinuities may reflect a difference between the parental AS genomes and that of the reference sequence, AS-WTSI, such that large regions of the genome that are linked by assembly (according to AS-WTSI) have, in our AS clone, undergone translocation to an unlinked location. If either the donor, or destination location exhibit different AJ proportions (e.g because of selection) then translocation will produce an apparent discontinuity (which would resolve itself if the translocation was incorporated into the assembly used as reference). For example, the AS-WTSI sequence used for this mapping (PlasmoDB v 6.3 Feb 2010) contained a number of instances where the assembly of a chromosome was ambiguous or ill-defined. For example, there was a break in the sequence assembly on chr12, and the AS-WTSI sequence defined a 'run' of 100 'N's at this location. This coincided with one of the discontinuities presented here (chr12D, Additional File 8). Subsequently, that assembly was modified (Sanger Sept 2009 (paradoxically, a later assembly than the PlasmoDB assembly defined above)): sequences to the right of this discontinuity were moved to the 'bin' of non-assigned sequences (assembled as one contig). This removes this discontinuity and shows that assembly inconsistencies would produce the patterns of data observed here. We therefore suggest that the AS genome assembly (in the strain used in our study) is likely to be different to that of the AS-WTSI genome reference sequence at a limited number of sites. These instances may reflect a divergence between the AS parasites used at WTSI and our laboratory, or indicate loci where assembly is yet to be optimised. In these cases, the genetic data presented here may indicate alternative assembly models. For example,

the final syntenic block on chr12 (now moved to 'bin') may be linked to the right hand end of chr11, and vice versa. However, we cannot always reject the possibility that, in some instances, inter-strain genome rearrangements (above) may contribute to the patterns observed.
