# Supplementary material for: Quantitative genome re-sequencing defines multiple mutations conferring chloroquine resistance in rodent malaria
Source: BMC Genomics. 2012 Mar 21;13:106. doi: 10.1186/1471-2164-13-106 (PMC3362770; doi:10.1186/1471-2164-13-106)
Supplement: Additional file 2 — (Table) Solexa whole-genome re-sequencing metrics. [file 1471-2164-13-106-S2.PDF]

Additonal File 2 Solexa whole-genome re-sequencing metrics

	AJ	AJ x AS-30CQ untreated	AJ x AS-30CQ CQ treated
**Numbers of reads sequenced**	38,719,648	33,254,128	33,110,368
**Mean -fold coverage**	103x	88x	88x
**Numbers of reads mapped**	36,247,350	31,268,716	31,416,153
**% reads mapped**	93.6%	94.0%	94.9%
**-fold coverage mapped**	x96	x83	x83
**Numbers of reads uniquely mapped**	35,597,299	30,683,918	30,845,976
**% reads uniquely mapped**	91.9%	92.3%	93.2%
**-fold coverage uniquely mapped**	x94	x82	x82
