# Supplementary figures and images for: Quantitative genome re-sequencing defines multiple mutations conferring chloroquine resistance in rodent malaria
Source: BMC Genomics. 2012 Mar 21;13:106. doi: 10.1186/1471-2164-13-106 (PMC3362770; doi:10.1186/1471-2164-13-106)

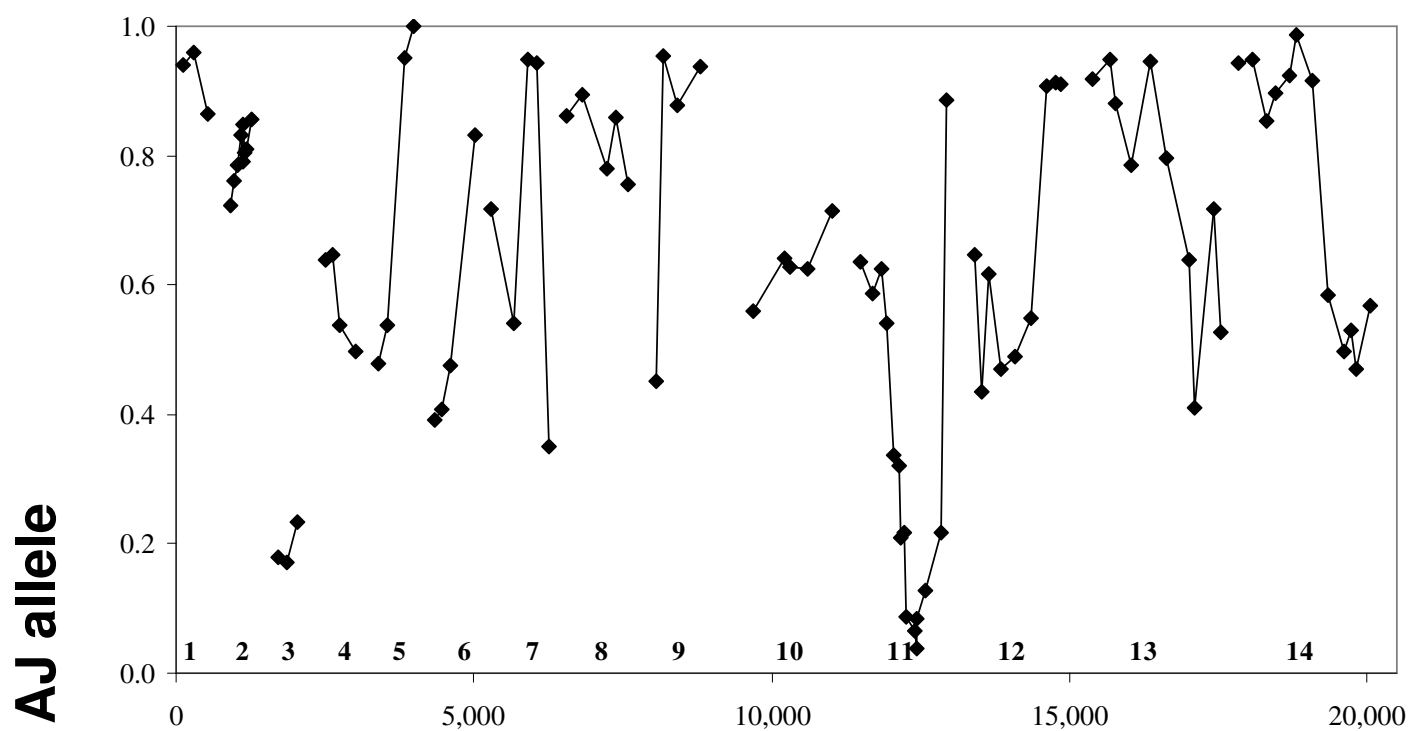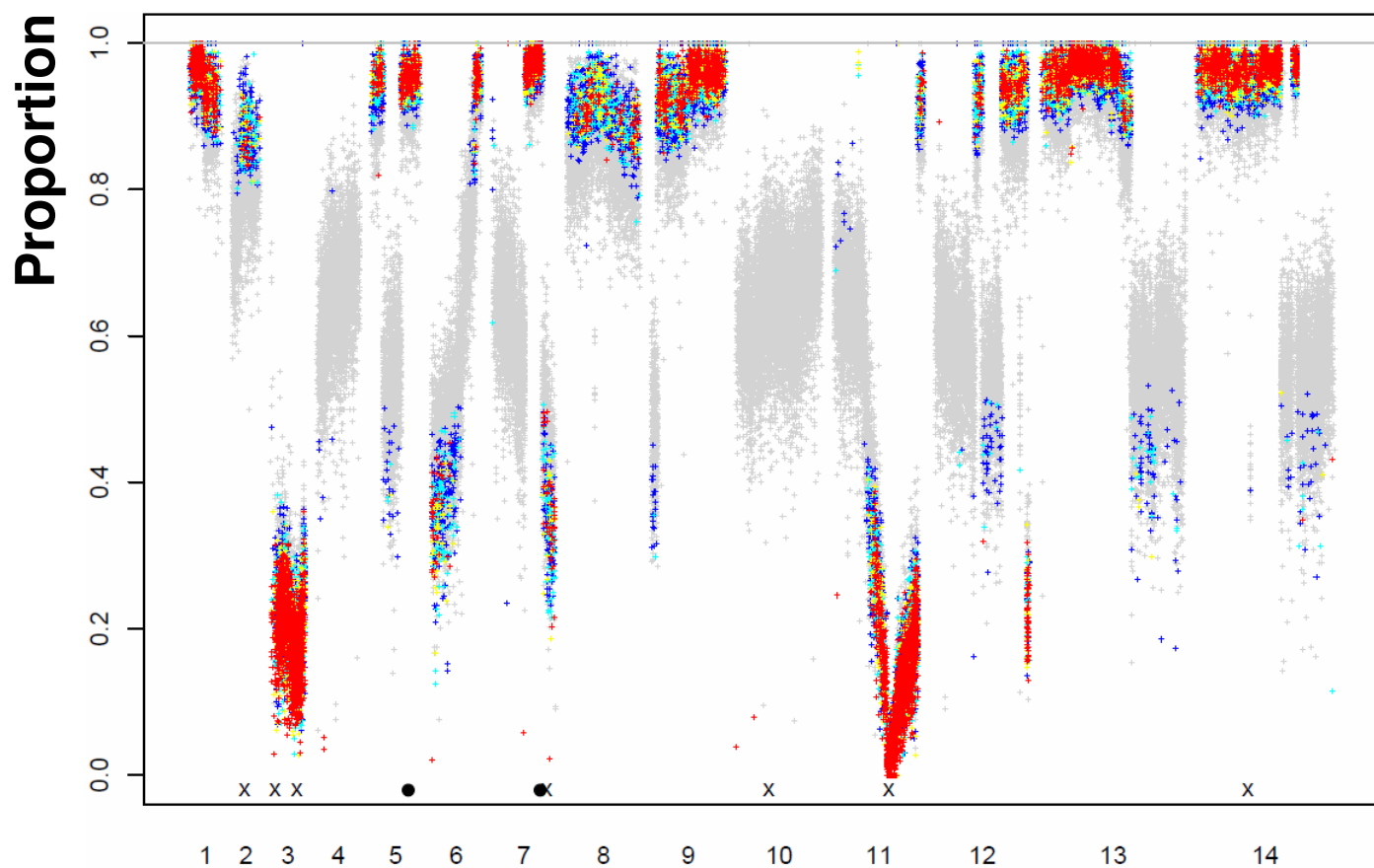

(approx) genome co-ordinate (nt) and/or chromosome no.

Supplement: Additional file 3 — (Figure) LGS-pyro v LGS-Illumina. Comparison of genome scans (LGS-pyro (top), LGS-Illumina (bottom)) show near perfect correspondence between the two methodologies. Vertical axis (linear) indicates proportion of AJ alleles in parasites surviving 3 mg CQ kg-1 day-1. Horizontal axis indicates chromosome number, top and bottom or genome co-ordinate (Kbase), top only. Position of mutations (AS-30CQ relative to AS-sens) are indicated at bottom of bottom panel (7 SNPs x, 2 deletions •). [file 1471-2164-13-106-S3.PDF]

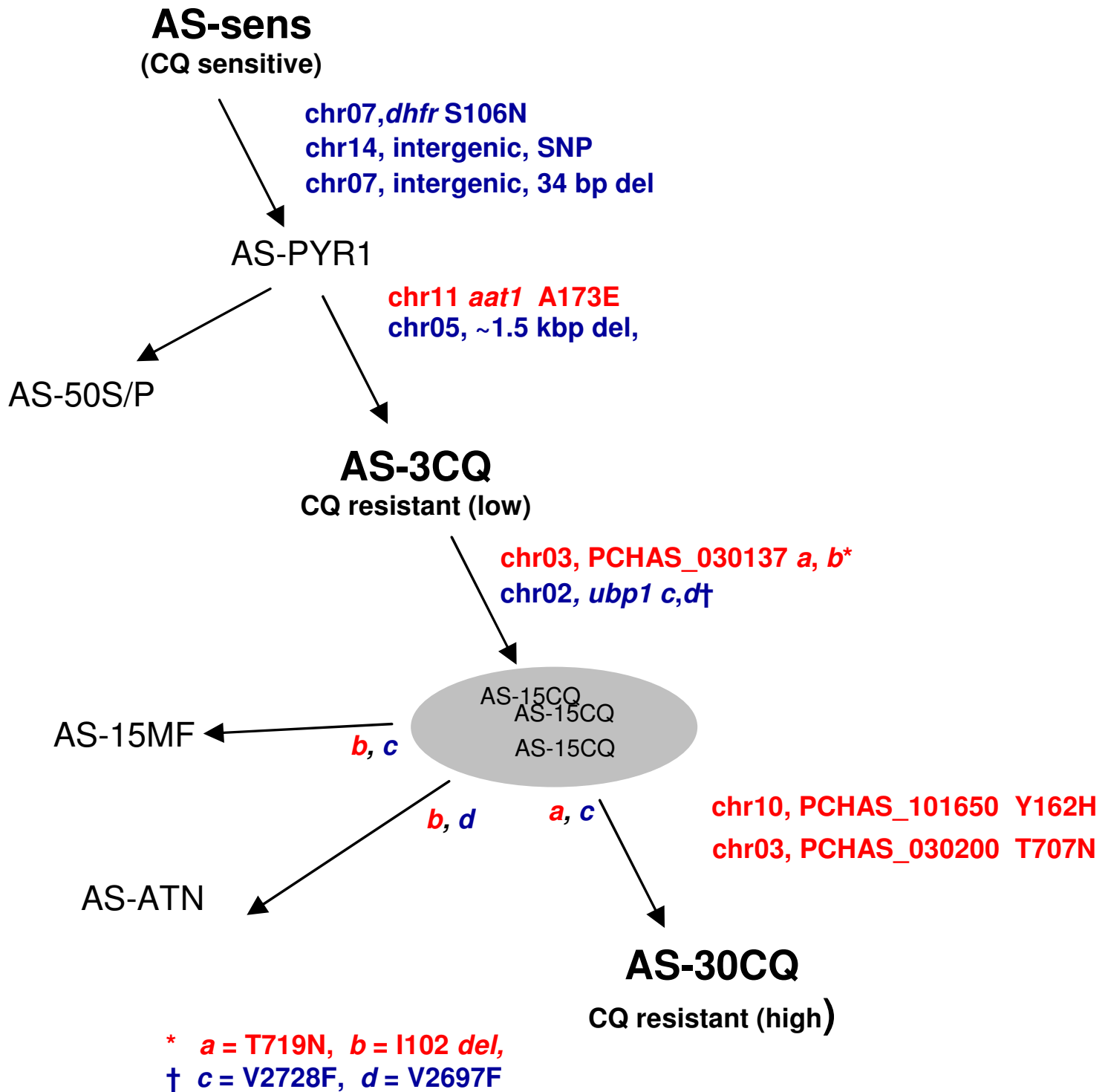

Supplement: Additional file 6 — (Figure) The appearance of mutations in the AS lineage. Mutations are described by chromosomal location, gene ID, specific amino acid change etc. Some were previously described [29,30] (blue). Novel mutations are identified here (red). For both PCHAS_030137 and ubp1, alternative mutations arising between AS-3CQ and AS-15CQ (and individually selected in AS-15MF [31,35] and AS-ATN [55] during mefloquine and artesunate selection, respectively) are defined (a-d). Refer to Additional File 1(section 3) for further details. [file 1471-2164-13-106-S6.PDF]
