# Supplementary material for: Quantitative genome re-sequencing defines multiple mutations conferring chloroquine resistance in rodent malaria
Source: BMC Genomics. 2012 Mar 21;13:106. doi: 10.1186/1471-2164-13-106 (PMC3362770; doi:10.1186/1471-2164-13-106)
Supplement: Additional file 4 — (Table) AS-30CQ Genome re-sequencing. Summary of all the mutations proposed in clone AS-30CQ (Additional File 1). Highlighted are mutations confirmed by di-deoxy sequencing (green), rejected mutations (red), a high confidence deletion (yellow) and low confidence mutations (orange). Read depth according to SSAHA2 is provided for SNPs. All quality scores for SNPs were according to SSAHA2. Small indel quality scores indicate the number of reads calling an indel divided by the total number of reads covering the indel. For large indels and CNVs, a comparative coverage was calculated as described (Methods section and Additional File 1). [file 1471-2164-13-106-S4.PDF]

# Additional File 4 AS-30CQ Genome re-sequencing

**Potential SNPs**

| Chr'some | Type | Analysis | Start (indels only) | End | reference base | Base in AS-30CQ | Read Depth in AS3-0CQ (SNPs only) | SSAHA Quality/CC | Confirmation of mutation by dideoxy sequencing | aa change | P. chabaudi Gene ID | Nearest gene ID (P. chabaudi) | P. falciparum orthologue |
|---|---|---|---|---|---|---|---|---|---|---|---|---|---|
| **2** | SNP | SSAHA/MAQ | | 216,954 | C | A | 72 | 99 | YES | V2728F | PCHAS_020720 | | *PFA0220w* |
| **3** | SNP | SSAHA/MAQ | | 70,553 | G | T | 61 | 99 | YES | T707N | PCHAS_030200 | | *None* |
| **3** | SNP | SSAHA/MAQ | | 474,123 | C | A | 57 | 99 | YES | T719N | PCHAS_031370 | | *PFB0675w* |
| **5** | SNP | SSAHA | | 681,914 | T | A | 4 | 6 | | | PCHAS_051910-20 | | *None* |
| **7** | SNP | SSAHA/MAQ | | 994,546 | G | A | 87 | 99 | YES | S106N | PCHAS_072830 | | *PFD0830w* |
| **10** | SNP | SSAHA/MAQ | | 634,932 | T | C | 37 | 99 | YES | Y162H | PCHAS_101550 | | *PF14_0279* |
| **11** | SNP | SSAHA/MAQ | | 996,332 | G | T | 111 | 99 | YES | A173E | PCHAS_112780 | | *PFF1430c* |
| **13** | SNP | SSAHA | | 113,002 | G | A | 4 | 8 | NO | | PCHAS_130280 | | *None* |
| **14** | SNP | SSAHA/MAQ | | 936,945 | T | G | 26 | 92 | YES | | | 5'-PCHAS_142600 | *PF08_0081* |
| **bin** | SNP | SSAHA | | 116,902 | A | C | 25 | 16 | | | PCHAS_000280 | | *None* |
| **bin** | SNP | SSAHA | | 167,611 | T | C | 4 | 15 | | | PCHAS_000460 | | *None* |
| **bin** | SNP | SSAHA | | 221,856 | T | G | 6 | 2 | | | PCHAS_000590 | | *None* |

**Potential Indels**

| Chr'some | Type | Analysis | Start | End | extent of region (bp) | comparative coverage | small indel quality index | Confirmation | P. chabaudi Gene ID | Nearest gene ID (P. chabaudi) |
|---|---|---|---|---|---|---|---|---|---|---|
| **1** | Deletion | SSAHA | 13,917 | 13,917 | 1 | | 20/38 | | | PCHAS_010040-5' |
| **1** | Deletion | SSAHA | 19,767 | 19,767 | 1 | | 28/50 | | | PCHAS_010050-3' |
| **1** | Insertion | SSAHA | 203,105 | 203,105 | 1 | | 10/19 | | | 5'-PCHAS_010530 |
| **1** | indel | SSAHA | 581,324 | 581,398 | 75 | 0.02 | | | | PCHAS_011540-5' |
| **2** | Insertion | SSAHA | 32 | 32 | 1 | | 2/3 | | | 3'-PCHAS_020010 |
| **3** | Deletion | SSAHA | 350,001 | 350,001 | 1 | | 2/4 | | | 5'-PCHAS_031010 |
| **4** | Deletion | SSAHA | 104,874 | 104,874 | 1 | | 14/16 | | | PCHAS_040280-5' |
| **4** | indel | SSAHA | 793,932 | 793,981 | 50 | 0.17 | | | | PCHAS_042080-5' |
| **5** | Insertion | SSAHA | 311,828 | 311,828 | 1 | | 26/30 | | PCHAS_050740 | |
| **5** | Deletion | SSAHA | 410,773 | 410,773 | 1 | | 2/3 | | PCHAS_050980 | |
| **5** | indel | SSAHA/MAQ | 683,724 | 684,989 | 1,266 | 0.24 | | tbc | PCHAS_051910-20 | |
| **6** | indel | SSAHA | 372,860 | 372,883 | 24 | 0.00 | | | PCHAS_060950 | |
| **7** | indel | SSAHA | 169 | 181 | 13 | 0.00 | | | | 5'-PCHAS_070010 |
| **7** | indel | SSAHA | 13,193 | 13,209 | 17 | 0.18 | | | PCHAS_070040 | |
| **7** | indel | SSAHA/MAQ | 876,907 | 876,921 | 15 | 0.19 | | 34bp deletion | | PCHAS_072420-3' |
| **7** | Insertion | SSAHA | 910,274 | 910,274 | 1 | | 2/2 | | | 3'-PCHAS_072530 |
| **7** | Deletion | SSAHA | 910,327 | 910,328 | 2 | | 2/3 | | | 3'-PCHAS_072530 |
| **7** | Insertion | SSAHA | 910,341 | 910,341 | 1 | | 6/7 | | | 3'-PCHAS_072530 |
| **8** | Insertion | SSAHA | 1,184,534 | 1,184,534 | 1 | | 2/3 | | | 3'-PCHAS_083190 |
| **9** | Deletion | SSAHA | 911,419 | 911,419 | 1 | | 6/11 | | | 3'-PCHAS_092680 |
| **9** | Deletion | SSAHA | 1,301,305 | 1,301,307 | 3 | | 13/25 | | | 3'-PCHAS_093730 |
| **10** | Deletion | SSAHA | 290,661 | 290,661 | 1 | | 43/47 | NO | | 3'-PCHAS_100680 |
| **10** | Deletion | SSAHA | 718,122 | 718,122 | 1 | | 57/71 | | | PCHAS_101800-3' |
| **11** | Insertion | SSAHA | 356,249 | 356,249 | 1 | | 2/3 | | | 5'-PCHAS_110990 |
| **11** | Deletion | SSAHA | 760,507 | 760,507 | 1 | | 24/24 | NO | PCHAS_112150 | |
| **11** | indel | SSAHA | 788,143 | 788,159 | 17 | 0.23 | | | | 5'-PCHAS_112230 |
| **11** | Deletion | SSAHA | 836,567 | 836,567 | 1 | | 36/45 | | PCHAS_112370 | |
| **12** | Insertion | SSAHA | 142,723 | 142,723 | 1 | | 4/8 | | | 5'-PCHAS_120380 |
| **12** | Deletion | SSAHA | 203,419 | 203,419 | 1 | | 11/18 | | PCHAS_120610 | |
| **13** | Deletion | SSAHA | 872,812 | 872,812 | 1 | | 5/8 | | | 5'-PCHAS_132230 |
| **13** | Insertion | SSAHA | 879,482 | 879,482 | 1 | | 29/37 | | | 5'-PCHAS_132250 |
| **13** | Deletion | SSAHA | 1,188,222 | 1,188,222 | 1 | | 21/30 | | PCHAS_133100 | |
| **13** | Deletion | SSAHA | 1,792,799 | 1,792,799 | 1 | | 3/5 | | | 3'-PCHAS_134780 |
| **13** | Insertion | SSAHA | 2,115,249 | 2,115,249 | 1 | | 5/9 | | | 3'-PCHAS_135780 |
| **13** | indel | SSAHA | 2,307,750 | 2,307,761 | 12 | 0.25 | | | | PCHAS_136280-5' |
| **14** | Insertion | SSAHA | 82,779 | 82,779 | 1 | | 2/2 | | | 5'-PCHAS_140200 |
| **14** | CNV | SSAHA | 1,595,078 | 1,595,331 | 254 | 3.20 | | | | 3'-PCHAS_144430 |
| **14** | indel | SSAHA | 2,015,046 | 2,015,060 | 15 | 0.25 | | | PCHAS_145480 | |
| **bin** | Insertion | SSAHA | 798 | 798 | 1 | | 12/24 | | | 5'-PCHAS_000010 |
| **bin** | Deletion | SSAHA | 35,739 | 35,739 | 1 | | 2/2 | | | 5'-PCHAS_000110 |
| **bin** | Deletion | SSAHA | 262,255 | 262,255 | 1 | | 9/9 | | | 5'-PCHAS_000700 |

Summary of all the mutations proposed in clone AS-30CQ (Additional File 1). Highlighted are mutations investigated by di-deoxy sequencing (green, confirmed; red, rejected) as well as a high confidence deletion (yellow) and other low confidence mutations (orange). Read depth according to SSAHA2 is provided for SNPs. All quality scores for SNPs were according to SSAHA2. Small indel quality scores indicate the number of reads calling an indel divided by the total number of reads covering the indel. For large indels and CNVs, a comparative coverage was calculated as described in the Additional File 1.
