# Supplementary material for: Quantitative genome re-sequencing defines multiple mutations conferring chloroquine resistance in rodent malaria
Source: BMC Genomics. 2012 Mar 21;13:106. doi: 10.1186/1471-2164-13-106 (PMC3362770; doi:10.1186/1471-2164-13-106)
Supplement: Additional file 5 — (Table) Genome-wide analysis of % of bases with read-coverage ≥ 10. [file 1471-2164-13-106-S5.PDF]

Additional File 5 Genome-wide analysis of % of bases with read-coverage ≥10

Chromosome	% coverage ≥ 10
Chr01	96.16%
Chr02	98.92%
Chr03	96.32%
Chr04	97.48%
Chr05	97.71%
Chr06	98.04%
Chr07	96.61%
Chr08	99.27%
Chr09	99.39%
Chr10	98.11%
Chr11	98.78%
Chr12	98.88%
Chr13	98.74%
Chr14	98.84%
**Total (chrs only)**	**98.31%**
BIN	71.69%
PCHAS_031370 +/-200 kb on chr03	99.28%
AAT1 +/-200 kb on chr11	99.27%
