# Supplementary material for: Quantitative genome re-sequencing defines multiple mutations conferring chloroquine resistance in rodent malaria
Source: BMC Genomics. 2012 Mar 21;13:106. doi: 10.1186/1471-2164-13-106 (PMC3362770; doi:10.1186/1471-2164-13-106)
Supplement: Additional file 7 — (Figure) Chromosome 7 - 34 bp deletion. Close to the 3' end of gene PCHAS_072420, an alignment of nucleotide sequences for reference genome sequence (AS-WTSI), the sensitive AS lineage progenitor (AS-sens) and the drug resistant mutants AS-PYR, AS-3CQ, AS-30CQ and AS-ART is shown. Symbols: -,34 bp deletion in AS-PYR and subsequent clones (wrt nt 197 - 230 (AS-WTSI arbitrary numbering) inclusive), 15 bp deletion in AS strains (wrt nt 306 - 320 (AJ arbitrary numbering) inclusive); *, nucleotides identical in all clones and strains investigated here (note high frequency of AS/AJ SNPs). 3' end of coding sequence of gene PCHAS_072420 is indicated (upper case, green highlighting); intron (lower case, grey highlighting), 3'-UT and intergenic region indicated (lower case); termination codon (red). Repetitive sequences which may mediate the 34 bp and 15 bp deletions are indicated (yellow) in individual representative clones. [file 1471-2164-13-106-S7.PDF]

```
       PCHAS_072420intron intron intron intron intron intron intron intron i
AS-WTSI         CAGgttcgttttgtacataccttattagtgtaaccatgtgtatgtcatactactttaata 60
AS-SENS         CAGgttcgttttgtacataccttattagtgtaaccatgtgtatgtcatactactttaata 60
AS-PYR          CAGgttcgttttgtacataccttattagtgtaaccatgtgtatgtcatactactttaata 60
AS-3CQ          CAGgttcgttttgtacataccttattagtgtaaccatgtgtatgtcatactactttaata 60
AS-30CQ         CAGgttcgttttgtacataccttattagtgtaaccatgtgtatgtcatactactttaata 60
AS-ART          CAGgttcgttttgtacataccttattagtgtaaccatgtgtatgtcatactactttaata 60
AJ              CAGgttcgttttgtacatcccttattagggtagccatgtgcatgtcatactaatttaata 60
                ****************** ********* *** ******* *********** *******

                ntron intron intron intron intron inPCHAS_072420 PCHAS_07242
AS-WTSI         tgatacaacgtatttgtgactattttccttttgcagGCTTTACAAATATACTATCTGCTT 120
AS-SENS         tgatacaacgtatttgtgactattttccttttgcagGCTTTACAAATATACTATCTGCTT 120
AS-PYR          tgatacaacgtatttgtgactattttccttttgcagGCTTTACAAATATACTATCTGCTT 120
AS-3CQ          tgatacaacgtatttgtgactattttccttttgcagGCTTTACAAATATACTATCTGCTT 120
AS-30CQ         tgatacaacgtatttgtgactattttccttttgcagGCTTTACAAATATACTATCTGCTT 120
AS-ART          tgatacaacgtatttgtgactattttccttttgcagGCTTTACAAATATACTATCTGCTT 120
AJ              tgatacaacttacttgtgaatattttccttttgcagGCTCTACAAATATACTATCTGCTT 120
                ********* ** ****** ******************* ********************

                0 PCHAS_072420 PCHAS_072420 P
AS-WTSI         CCTACCAGCAAATATTGATGTGCCAATAAttaaatgtgatgggactattgaacagtttgt 180
AS-SENS         CCTACCAGCAAATATTGATGTGCCAATAAttaaatgtgatgggactattgaacagtttgt 180
AS-PYR          CCTACCAGCAAATATTGATGTGCCAATAAttaaatgtgatgggactattgaacagtttgt 180
AS-3CQ          CCTACCAGCAAATATTGATGTGCCAATAAttaaatgtgatgggactattgaacagtttgt 180
AS-30CQ         CCTACCAGCAAATATTGATGTGCCAATAAttaaatgtgatgggactattgaacagtttgt 180
AS-ART          CCTACCAGCAAATATTGATGTGCCAATAAttaaatgtgatgggactattgaacagtttgt 180
AJ              CCTACCAGCAAATATTGATGTGCCAATAAttaaatgtgatgggactattgaacagcttgt 180
                ******************************************************* ****

AS-WTSI         tatttactatgggatggattattaaccagtttgttatttactatgggatgaaataaggag 240
AS-SENS         tatttactatgggatggattattaaccagtttgttatttactatgggatgaaataaggag 240
AS-PYR          tatttactatgggatg----------------------------------aaataaggag 206
AS-3CQ          tatttactatgggatg----------------------------------aaataaggag 206
AS-30CQ         tatttactatgggatg----------------------------------aaataaggag 206
AS-ART          tatttactatgggatg----------------------------------aaataaggag 206
AJ              tatttactatgggatggattattaaccagtttgttatttcctatgggatgaaataaggag 240
                ****************                                  **********

AS-WTSI         gaccccaagcatggcatatataaacacaattatgtggattggcaaaccgtaatttattcc 300
AS-SENS         gaccccaagcatggcatatataaacacaattatgtggattggcaaaccgtaatttattcc 300
AS-PYR          gaccccaagcatggcatatataaacacaattatgtggattggcaaaccgtaatttattcc 266
AS-3CQ          gaccccaagcatggcatatataaacacaattatgtggattggcaaaccgtaatttattcc 266
AS-30CQ         gaccccaagcatggcatatataaacacaattatgtggattggcaaaccgtaatttattcc 266
AS-ART          gaccccaagcatggcatatataaacacaattatgtggattggcaaaccgtaatttattcc 266
AJ              gaccccaagtatggcatacatatatacaattgtgtggattggcaaaccggaatttatttc 300
                ********* ******** *** * ****** ***************** ******** *

AS-WTSI         cacat---------------ttgttttaaaaggttgaacttttataacgtagattataca 345
AS-SENS         cacat---------------ttgttttaaaaggttgaacttttataacgtagattataca 345
AS-PYR          cacat---------------ttgttttaaaaggttgaacttttataacgtagattataca 311
AS-3CQ          cacat---------------ttgttttaaaaggttgaacttttataacgtagattataca 311
AS-30CQ         cacat---------------ttgttttaaaaggttgaacttttataacgtagattataca 311
AS-ART          cacat---------------ttgttttaaaaggttgaacttttataacgtagattataca 311
AJ              cacattcattcccacatgatttgttttaaaaggttgaacttttataacgtagattataca 360
                *****               ****************************************

AS-WTSI         aattgaaaagaaaaatatataatggctatctaaaatgttgtcacagcctttatacg    401
AS-SENS         aattgaaaagaaaaatatataatggctatctaaaatgttgtcacagcctttatacg    401
AS-PYR          aattgaaaagaaaaatatataatggctatctaaaatgttgtcacagcctttatacg    367
AS-3CQ          aattgaaaagaaaaatatataatggctatctaaaatgttgtcacagcctttatacg    367
AS-30CQ         aattgaaaagaaaaatatataatggctatctaaaatgttgtcacagcctttatacg    367
AS-ART          aattgaaaagaaaaatatataatggctatctaaaatgttgtcacagcctttatacg    367
AJ              aattgaaaagaaaaatatatgatggctatctaaaatgttgtcacagcctttatacg    416
                ******************** ***********************************
```
