# Supplementary material for: Quantitative genome re-sequencing defines multiple mutations conferring chloroquine resistance in rodent malaria
Source: BMC Genomics. 2012 Mar 21;13:106. doi: 10.1186/1471-2164-13-106 (PMC3362770; doi:10.1186/1471-2164-13-106)
Supplement: Additional file 8 — (Table) Discontinuity co-ordinates. Co-ordinates represent nucleotide position on chromosome relative to the AS-WTXI sequence assembly (Sanger Sept 2009); nd, cannot be determined. [file 1471-2164-13-106-S8.PDF]

Additional File 8 Discontinuity co-ordinates

chromosome number	discontinuity letter (id)	SNP to left of discontinuity	SNP to right of discontinuity	interval (nucleotides)
5	A	218,871	219,155	284
	B	551,082	552,390	1,308
7	A	606,123	606,825	702
	B	917,636	917,826	190
9	A	112,964	113,833	869
11	C	1,531,841	1,532,005	164
12	A	729,852	730,065	213
	B	865,332	866,098	766
	C	1,224,431	1,224,680	249
	D	1,682,022	bin:359,580	nd
13	A	1,644,125	1,644,854	729
14	A	1,539,100	1,539,197	97
	B	1,752,282	1,753,404	1,122
	C	1,836,461	1,836,746	285

Co-ordinates represent nucleotide position on chromosome relative to the AS-WTSI sequence assembly (Sanger Sept 2009); nd, cannot be determined.
