# Supplementary material for: Quantitative genome re-sequencing defines multiple mutations conferring chloroquine resistance in rodent malaria
Source: BMC Genomics. 2012 Mar 21;13:106. doi: 10.1186/1471-2164-13-106 (PMC3362770; doi:10.1186/1471-2164-13-106)
Supplement: Additional file 9 — (Table) Primers used. These oligonucleotide primers were used to confirm the predicted mutations in AS lineage. Pairs of primers marked with * were also used for proportional sequencing. [file 1471-2164-13-106-S9.PDF]

Additional File 9 Primers used

Chromosome	Mutation	Forward primer	Reverse primer
2	PCHAS_020720 V2728F *	CTTTTGTTTCTCATAATCCAG	GGTTTAGGTTTTGCAAAGAA
3	PCHAS_030200 T707N	CTGGAACAAGCACAACAGC	TTCATTGGTGCTCCTGTATC
3	PCHAS_031370 T719N *	GAAACTAACAGGCCCACAC	TAGCACAATGGGAAAAATTAG
7	PCHAS_072830 S106N	TTTTAAGGGACTTGGGAATG	ATAAATCTTCATTGATATCTGG
7	34bp del	CGTATAAAGGCTGTGACAAC	CAGGTTCGTTTTGTACATACC
10	PCHAS_101550 Y162H	GAGACGATGAGTGTTTACTTTCC	AATAGTACCTGTGTTGTTAGTCGC
11	PCHAS_112780 A173E *	GGAGTGATAAGAATGATAATATGAGG	GTGTTCAAGTGCTGACATGC
14	intergenic SNP	CTTCACTATAAATATGCTGTTC	CATGCCGATACTTATTATATGC

These oligonucleotide primers were used to confirm the predicted mutations in AS lineage. Pairs of primers marked with * were also used for proportional sequencing.
